# Galvanostatic cycling of a micron-sized solid-state battery: Visually linking void evolution to electrochemistry

**DOI:** 10.1126/sciadv.adt4666

**Published:** 2025-04-04

**Authors:** Haowen Gao, Chen Lin, Yuanpeng Liu, Jiashun Shi, Bowen Zhang, Zhefei Sun, Zhao Li, Yu Wang, Menghao Yang, Yong Cheng, Ming-Sheng Wang

**Affiliations:** ^1^ State Key Laboratory of Physical Chemistry of Solid Surfaces, College of Materials, Xiamen University, Xiamen, 361005, China.; ^2^ Sino-French Institute of Nuclear Engineering and Technology, Sun Yat-Sen University, Zhuhai, 519000, China.; ^3^ National Key Laboratory of Science and Technology on Advanced Composites in Special Environments and Center for Composite Materials and Structures, Harbin Institute of Technology, Harbin, 150080, China.; ^4^ School of Materials Science and Engineering, Tongji University, Shanghai, 201804, China.

## Abstract

The formation of interface voids, peculiar to the solid-solid contact between metal anodes and solid electrolytes (SEs), has become a fundamental obstacle for developing practical lithium metal solid-state batteries (SSBs). Addressing this issue requires the operando observation of void evolution with high spatio-temporal resolution and the direct linkage of voids to solid-state electrochemistry. Here, we present such an attempt by visualizing both the stripping and plating interfaces of a micron-sized SSB cycled in galvanostatic mode in a transmission electron microscope. Various voltage responses in the charge/discharge curves are well correlated to the nucleation, growth, and refilling of single voids. Notably, two distinct modes of Li stripping, namely, void-growth stripping and void-free stripping, are experimentally identified. We unveil the roles of stack pressure and current density on void evolutions, which suggests a mechanism of void suppression without involving plastic deformation of Li metal. Furthermore, Li|SE|Li symmetric SSBs enabling repeated void-free cycling without stack pressure are in situ demonstrated.

## Teaser

The smallest solid-state batteries are in situ constructed in an electron microscope to visualize the Li metal anode evolutions.

## INTRODUCTION

Solid-state batteries (SSBs) with lithium metal anodes have shown great promise for achieving high energy density and enhanced safety (*1*–*3*). However, the development of SSBs is still severely hindered by the interface issues between Li and solid electrolytes (SEs), such as Li filament growth and void formation (*4*–*11*). These two major problems occur at the Li/SE interface during Li deposition and dissolution, respectively. SEs that form no or passivating interphases with Li metal are often vulnerable to Li penetration, leading to a short circuit and even safety hazard (*6*, *9*, *12*–*14*). Compared to Li penetration, the formation of voids is actually more prevalent for SEs, regardless of their (electro)chemical stability against Li metal and the properties of the formed interphases (*15*–*20*). The loss of contact caused by void formation can result in impedance increase and severe performance degradation (*8*, *17*, *18*). Moreover, the remaining contact area endures a much higher local current density, which carries the risk of triggering Li metal penetration during subsequent Li deposition (*5*, *9*). Thus, unlike liquid electrolyte systems, the void formation peculiar to solid-solid interfaces is a more fundamental issue for governing the electrochemical behavior and stability at Li/SE interfaces.

The presence of voids at the cycled Li/SE interfaces is usually confirmed through postmortem morphological imaging by, e.g., electron or optical microscopies (*18*, *21*, *22*). However, in-depth mechanistic understanding of void evolution requires its direct observation at microscopic scale, which poses a great challenge because of the buried nature of Li/SE interface. To date, only the techniques that can penetrate materials may provide the possibility for operando visualizing the interface evolutions, such as x-ray computed tomography (XCT). Recently, XCT was first used to image the Li/SE interface during battery cycling, but only the voids that have grown sufficiently large (at least microns in size) can be resolved due to the limited spatial resolution of XCT (*5*, *16*, *23*). Some critical questions as to how a void nucleates, grows, and vanishes (under stack pressure or upon Li replating) are yet to be clarified. Therefore, operando imaging methods with high spatio-temporal resolution capable of probing nano/atomic-scale information are highly desired for investigating the void formation and Li/SE interfacial evolutions.

The formation of voids can also be identified indirectly by electrochemical methods (*7*, *8*, *17*, *18*). For example, in galvanostatic plating-stripping experiments, the prominent increase in polarization voltage is commonly correlated to the interface deterioration, especially the contact loss of the interface at the dissolution side. However, such methods would be easy to misinterpret the linkage between void evolution and electrochemistry due to the lack of direct observation of the interface dynamics. Besides, the electrochemical results obtained by conventional cell tests should be a collective response to all the morphological and electrochemical changes across the entire Li/SE interface, including the evolution of a large number of voids and other interfacial processes (*18*, *24*). Ideally, the voltage response is expected to be directly linked to the evolution of a single void, which should be highly valuable for correctly understanding the detailed mechanism of void evolution and the related electrochemistry. Obviously, to gain such an experimental insight would be extremely difficult and has so far never been achieved.

Here, micron-sized SSBs based on a single Li_6.4_La_3_Zr_1.4_Ta_0.6_O_12_ (LLZO) particle are constructed inside a transmission electron microscope (TEM), which allows in situ observation of the Li/SE interface evolution from a cross-sectional perspective, as well as the direct linkage between individual voids to electrochemistry under galvanostatic cycling. Void formation and refilling are clearly visualized, and the contact loss/rebuild is well linked to the synchronous voltage response. We also show the direct evidence that surface defects [e.g., grain boundaries (GBs)] and contaminations are the preferred sites for void nucleation. Notably, Li can be freely stripped layer-by-layer (LBL) without the formation of voids, exhibiting no voltage polarization. Such void-free Li stripping is achievable if the mechanical resistance that hinders the retraction of Li metal toward SE is overcome. We also reevaluate the role of stack pressure in void suppression: driving the drift of Li anode and maximizing the LBL stripping at the interface. Furthermore, two ways to achieve repeated void-free cycling of symmetric Li|LLZO|Li cells without stack pressure are demonstrated, which was enabled by a floating LLZO particle or a flexible nanotube current collector (CC).

## RESULTS

### Li stripping with and without void formation at Li/LLZO interface

**Fig. 1.**
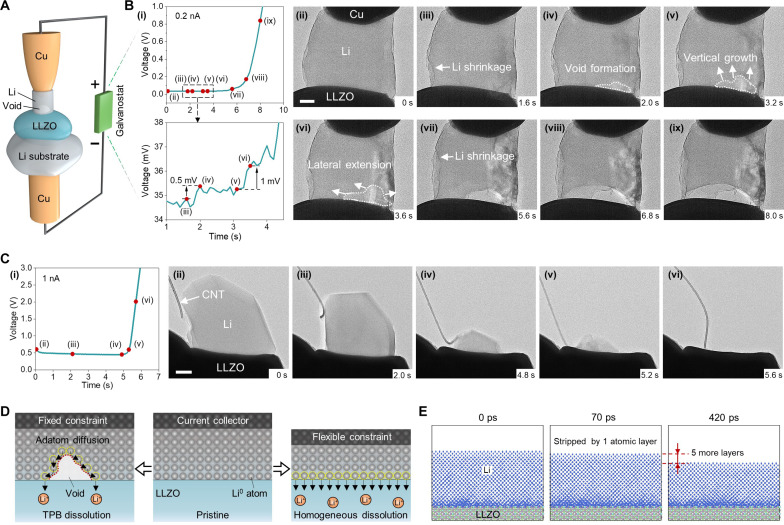
Galvanostatic stripping of Li metal with and without void formation. (**A**) Schematic of the microscale SSB setup cycled in galvanostatic mode. (**B**) (i) Recorded voltage trace of Li stripping at 0.2 nA, with the enlarged profile at the early stripping stage (below) and (ii to ix) the corresponding sequential TEM snapshots of Li stripping with the mechanical resistance from a rigid Cu CC. (**C**) (i) Voltage profile of Li dissolution at 1 nA with a soft CNT as CC and (ii to vi) the corresponding sequential TEM snapshots. (**D**) Schematic illustration of two stripping modes, i.e., void-growth stripping under fixed constraint (left) and homogeneous stripping with flexible constraint (right). (**E**) Snapshots of MD simulations of homogeneous Li stripping. Scale bars, 200 nm.

The in situ Li stripping experiments started with a microscale anode-free SSB setup, where a LLZO particle was semisubmerged in a Li metal electrode and the opposite copper probe was used as a CC (see Materials and Methods and fig. S4 for details). A certain amount of Li metal was first in situ deposited between the Cu probe and LLZO (Fig. 1, A and Bii). Then, the applied current was reversed to initiate the stripping process at 0.2 nA, and the corresponding voltage curve was recorded (Fig. 1Bi). The Li metal began to dissolve at its sidewall (indicated in Fig. 1Biii), whereas the intimate contact at the Li/LLZO interface remains unchanged. Previous models predicted a critical dissolution current density of 10 to 100 μA cm^−2^ based on bulk vacancy diffusion, which is far below the stripping current density of this case (~18 mA cm^−2^) and the practically required current densities (several milliamperes per square centimeter) (*8*, *25*). Therefore, adatom diffusion at the surface (terrace-ledge-kink model), much faster than bulk diffusion, is reasonably considered as the major pathway for mass transport in Li metal (*26*–*28*). In this path, Li atoms migrate along the surface toward the triple-phase boundary (TPB) (where the Li metal, LLZO, and vacuum meet), where Li^0^ is oxidized to Li^+^ and enters LLZO.

As the Li metal shrinkage slowed down at the sidewall, a void appeared at the Li/LLZO interface (Fig. 1Biv), resulting in a small area of contact loss and a slight increase of 0.5 mV in the polarization voltage (the zoom-in view of the curve in Fig. 1Bi). Meanwhile, the void surface provides a new path for the Li transport required for the galvanostatic stripping, and adatom diffusion still dominated the mass transport process. Subsequently, the void grew in the vertical direction (Fig. 1Bv) but did not bring obvious change in voltage. Instead, as the void extended laterally causing further contact loss (Fig. 1Bvi), the voltage showed another increase of 1 mV. The void grew three dimensionally via adatom diffusion, often companied with the extension of TPB that reduces the contact area. When a large portion of contact area was lost, the polarization voltage began to rise obviously (Fig. 1B, vii and viii). Meanwhile, the Li crystal on its left side underwent further dissolution (marked in Fig. 1Bvii), indicating that the surface diffusion on this side was still active. Eventually, the Li metal at the contact was almost completely depleted (Fig. 1Bix), leading the polarization voltage to quickly increase and reach the cutoff level (movie S1). We also performed control experiments with the electron beam blanked off, which demonstrated that the imaging beam has no obvious influence on the Li stripping behavior and the corresponding polarization voltage curve (fig. S5).

Our results show that the battery polarization can be linked to the loss of contact. However, in the early stage of void growth (e.g., Fig. 1B, iv to vi), the voltage increase upon contact loss is not obvious, probably because the formed void surface also introduces new diffusion paths inside Li. In some sense, void formation helps to maintain the constant stripping current. The problem is that only a small fraction of Li metal can be stripped before the severe polarization (e.g., ~8% in the case of Fig. 1B). In other words, the stripping current associated with void formation is not sustainable.

Therefore, we next explored the possible way of stripping without void formation. As shown in Fig. 1C, a flexible carbon nanotube (CNT) was used as the CC. After the deposition of a Li crystal (fig. S6), we conducted a galvanostatic stripping test at a current of 1 nA and recorded the synchronous voltage response (Fig. 1Ci). Notably, the Li crystal was freely retracted and dissolved into the LLZO, maintaining an intimate contact throughout the stripping process (movie S2). Accordingly, the voltage remained quite stable (Fig. 1C, ii to iv) until the Li metal was close to its full depletion (Fig. 1Cv). Eventually, the polarization voltage experienced a sharp increase at the end of stripping (Fig. 1Cvi), and nearly 100% of Li metal was stripped before polarization.

A close examination shows that the Li metal dissolved homogeneously at the interface, which avoids the formation of voids. In this pattern, the whole Li metal experiences a relative displacement toward LLZO. Under a constant current load, the Li crystal in Fig. 1 (C, ii to v) was retracted at a speed ranging from 81 to 190 nm s^−1^ (varying with the contact area), corresponding to the current density of 60 to 141 mA cm^−2^. In other cases, the fast Li retraction enabled an even higher stripping current density of ~2 A cm^−2^ (fig. S7 and movie S13). Compared with adatom diffusion, such free retraction is more efficient for mass transport at Li metal side, especially at higher current density (fig. S8).

Thus, two distinct stripping patterns were identified, namely, void-growth stripping and void-free stripping, as schematically illustrated in Fig. 1D. In the void-growth stripping, adatom diffusion dominates the stripping process, and Li atoms dissolve into the LLZO mainly through the TPB. We therefore also term it as nonhomogeneous dissolution or TPB dissolution. For the void-free stripping, Li metal dissolved homogeneously at the entire interface, in a seemingly atomically LBL pattern. In Fig. 1E, figs. S9 and S10, and movies S3 and S4, molecular dynamic (MD) simulations give atomic-scale insight of the stripping interface. A disordered Li layer of about 1 nm is formed and maintained at the incoherent interface between the single-crystal Li and LLZO. The crystalline Li atoms become disordered LBL near the interface and then dissolve uniformly into LLZO, leading to continuous thinning of the Li anode. Thus, we alternatively term it as homogeneous dissolution or LBL stripping (for simplicity, the disordered Li layer is not illustrated in the schematics of Fig. 1D).

The difference in the stripping behaviors originates from the distinct mechanical constraints imposed by the Cu and CNT CCs. The rigid Cu probe usually forms strong adhesion with Li metal and hinders its motion toward the interface (fig. S11), whereas the CNT is much flexible and allows the free retraction of Li metal. It is worth noting that the mechanical resistance can come from not only the CC but also the Li_2_O shell (due to the residual oxygen in TEM). For the stripping with a CNT, the Li_2_O shell can also block the retraction of Li metal, leading to void formation at the interface or on the shell (fig. S12). This is especially true for the low-rate stripping, where the unstripped part of Li metal has sufficient time to form a thick Li_2_O shell on the surface (fig. S13).

**Fig. 2.**
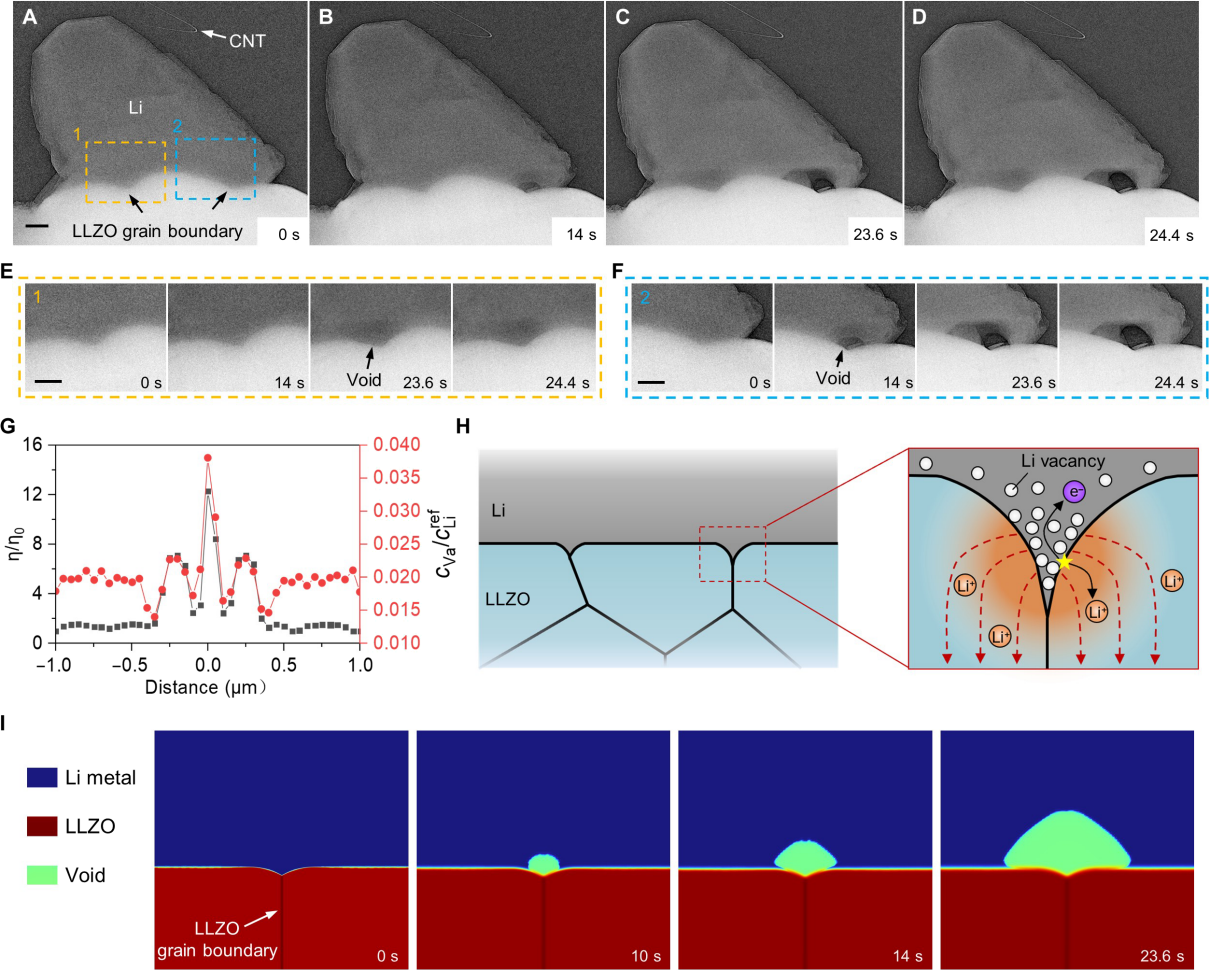
Void formation at the LLZO GB during Li stripping. (**A** to **D**) Time-resolved TEM images (contrast-inversed) of void nucleation and growth at the LLZO GB under a stripping current of 0.2 nA. (**E** and **F**) The enlarged images of sequential TEM snapshots of the region marked by the yellow and blue dashed rectangles in (A), respectively. (**G**) Distributions of normalized overpotential and relative vacancy concentration at the beginning of anodic loading along the Li/LLZO interface. The normalized overpotential means the ratio of potential (η) to the average potential of the interface (η_0_). The relative concentration of vacancy is the ratio of the vacancy concentration at the interface to the initial concentration of Li metal, and voids will form as the ratio reaches to 1. The origin of distance coordinate indicates the site of GBs. (**H**) Schematical illustration of the interface between Li and polycrystalline LLZO under anodic loading and the vacancy aggregation and current focusing at the GB. (**I**) Simulation images of void formation at the GB of LLZO. Scale bars, 200 nm.

We found the direct evidence that surface defects such as GBs and contaminants (e.g., Li_2_CO_3_) on LLZO can promote the local stripping and diffusion kinetics, leading to preferential void growth at these sites. Figure 2 presents a cross-sectional view of the stripping behavior of Li metal on polycrystalline LLZO composed by three grains. Figure 2 (A to D) shows the two GBs buried beneath Li metal before stripping. It can be observed that during the stripping process, a void preferentially formed and expanded at the right GB, along with the appearance of another void at the left GB. The sequential magnified views of the void nucleation and growth at the two GBs are depicted in Fig. 2 (E and F). Theoretical calculations in previous literatures indicate that the Li ionic conductivity of LLZO GBs is lower than that of its bulk phase (*29*–*31*). Therefore, we do not attribute the preferential formation of voids to the possible enhanced ion conduction at GBs. Instead, the geometric feature, i.e., the concave surface morphology at the GBs, may play a critical role in void formation at these locations. To further elucidate the mechanism, we established a phase-field model to simulate the Li dissolution process at a LLZO GB (see Fig. 2I). Figure 2G shows the simulation results of the normalized overpotential and the relative vacancy concentration along the Li/LLZO interface at the beginning of anodic loading. It indicates that the surface overpotential and vacancy concentration at the GB are considerably higher than the flat surface of the two adjacent grains. As schematically illustrated in Fig. 2H, the higher potential drives the faster dissolution, resulting in the higher local vacancy concentration. The enhancement in the overpotential suggests the electric field focusing caused by the concave surface geometry at the GB. Moreover, concave surfaces can provide favorable sites for vacancy aggregation and nucleation due to its lower energy barrier for heterogeneous nucleation (*32*, *33*), leading to void formation at the GB (Fig. 2I).

The surface contaminants can also facilitate the void formation. Figure S14 (A to D) illustrates the Li metal stripping process. As revealed by the enlarged images of the contaminated areas, voids preferentially form at the edges of the contaminants (fig. S14, E and F). As schematically illustrated in fig. S14G, the enhanced electric field at the edge of the contaminant leads to a higher local current density during stripping (*2*, *34*, *35*). Besides, the diffusion rate of Li atoms at the interface between the contaminant and Li metal may be faster than in the bulk of Li metal, thus leading to the faster stripping kinetics and preferential void formation. These results show that the defects and impurities at the surface of SEs are the preferential sites not only for Li nucleation during plating [as proved by previous findings (*27*, *36*, *37*)] but also for void formation during stripping.

### Void suppression via homogeneous Li stripping under stack pressure

**Fig. 3.**
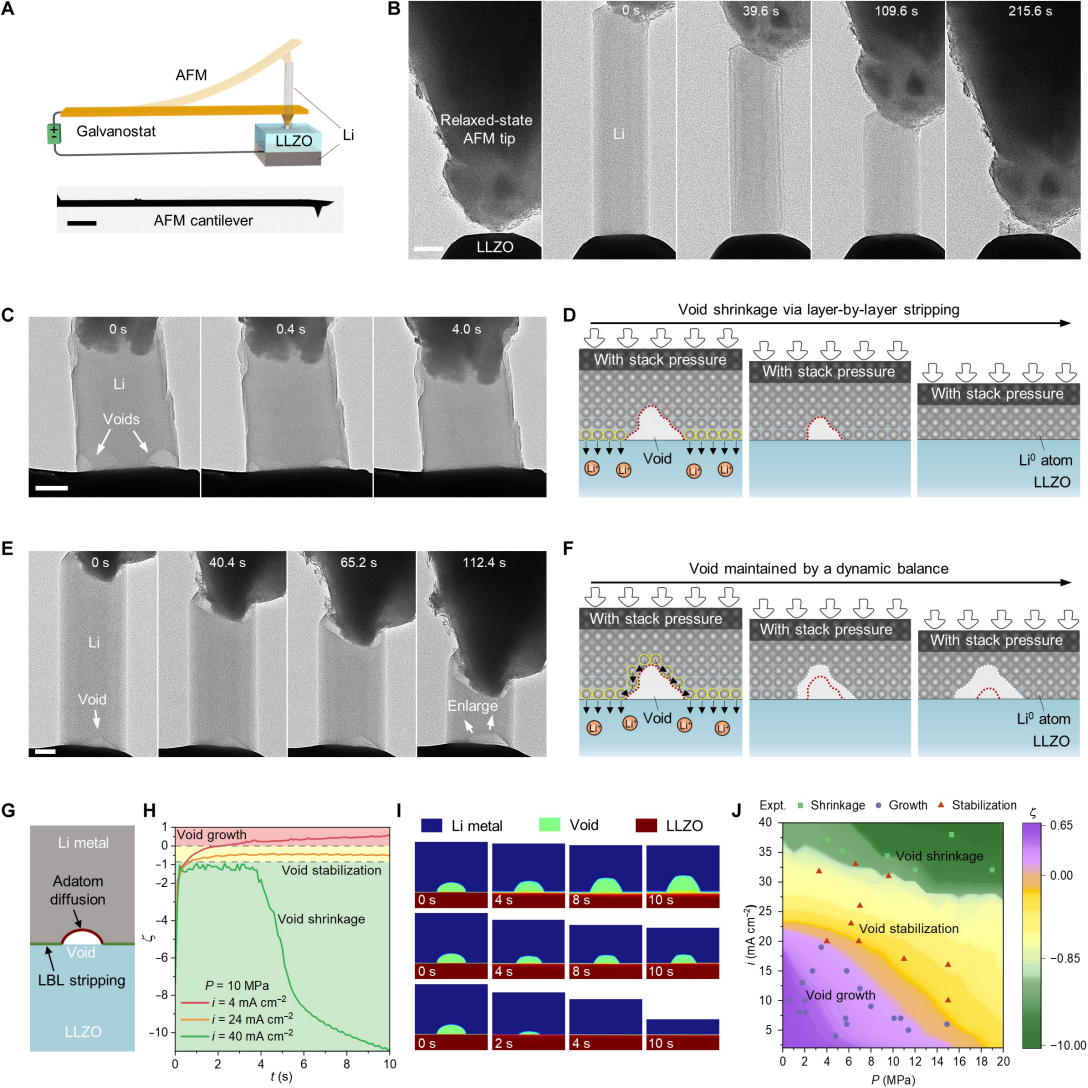
Li stripping under stack pressure exerted by an AFM cantilever. (**A**) Illustration of the TEM-AFM test setup. Scale bar, 25 μm. (**B**) Sequential TEM snapshots of Li stripping under compress stress. Scale bar, 500 nm. The spring constant of the AFM cantilever is 2.8 nN nm^−1^. (**C**) TEM images of the void elimination at Li/LLZO interface by increasing the stripping current density (from 3 to 60 mA cm^−2^) under stack pressure. Scale bar, 500 nm. (**D**) Schematic illustration of the void annihilated via LBL stripping. (**E**) Void growth suppression during stripping under stack pressure. Scale bar, 500 nm. (**F**) Schematic of the dynamic balance between adatom diffusion on void surface and LBL stripping at interface to maintain the void size. The red dashed lines outline the original void surface. (**G**) Model for phase-field simulation. (**H**) Simulation results of ζ as a function of time at different current densities under a stack pressure of 10 MPa, reflecting void growth, stabilization, and shrinkage, respectively. (**I**) The corresponding simulation images showing void growth, stabilization, and shrinkage, respectively. (**J**) *i-P* map revealing the void evolution regulated by the current density and stack pressure.

The above results suggest that to maintain void-free stripping, the mechanical resistance that impedes Li retraction should be overcome. In this regard, applying stack pressure, the commonly used strategy to suppress voids could be helpful. Therefore, an atomic force microscopy (AFM) cantilever was used as the CC to exert compressive stress on a grown Li whisker, as illustrated in Fig. 3A. After reversing the polarity of the applied current, the Li whisker began to dissolve at the Li/LLZO interface under stack pressure. Figure 3B and movie S5 show the LBL stripping of the Li whisker at a rate of 10 mA cm^−2^ under an initial stack pressure of ~14 MPa. The Li whisker was consistently shortened without forming voids under the decreasing pressure (the corresponding voltage curve is shown in fig. S15), with the Li_2_O debris crushed down from the Li whisker and accumulated at its root. Note that no visible plastic deformation was found in the Li whisker under compression. Previous researches also reported the yield strength of at least tens of megapascals for Li whiskers due to the size effect, much larger than the applied stress in our test (*38*–*40*). Hence, the observed void suppression effect under stack pressure is more likely the result from the rigid body displacement of Li anode rather than Li creep deformation (the widely accepted mechanism).

We also demonstrated that preexisting voids can be eliminated by the homogeneous stripping at the residual contact area (movie S6). As depicted in Fig. 3C, two voids were first intentionally induced during the stripping under low-rate and low-pressure conditions (see discussion in fig. S16). Under the initial pressure of ~4 MPa, we increased the current density to 60 mA cm^−2^. The Li metal at the remaining contact area dissolved LBL, gradually eliminating the two voids, as schematically illustrated in Fig. 3D (the disordered Li layer is not illustrated for simplicity). More similar cases are provided in figs. S17 and S18.

However, more frequently, we observed that once a void was formed, its growth could be restricted but not completely eliminated (figs. S19 and S20 and movie S7). As depicted in Fig. 3E, despite the shortening of the Li whisker caused by LBL stripping, the void was preserved throughout the stripping at 21 mA cm^−2^ and a decreasing pressure from ~7 MPa. This can be attributed to the presence of adatom diffusion on the void surface, which reached a dynamic equilibrium with LBL stripping. The former enlarges the void while the latter shrinks it, and these two opposite processes prevent the void from growing large in size, as illustrated in Fig. 3F. However, when the pressure decreased to ~2 MPa, the void began to enlarge (Fig. 3E, 112.4 s), indicating the role of higher pressure in aiding void suppression (also see fig. S21). Similarly, for the Li stripping at the GBs and contaminants, once the voids are formed, they cannot be completely eliminated due to the promoted the diffusion/stripping rate at these sites, even at a high current density (fig. S22). They can also be restricted in size by LBL stripping under stack pressure (fig. S22) or grow up (fig. S23), depending on the pressure/rate conditions.

These results suggest that higher pressure or current density favors the LBL stripping. To further quantitatively evaluate the effects of current density (*i*) and stack pressure (*P*) on void evolution during stripping, phase-field simulations were conducted. As illustrated in Fig. 3G, the geometry including a preexisting void at the interface was adopted in the simulation (see details in section S1 of the Supplementary Materials). The Li metal undergoes LBL stripping at the contact region (green line), and the red line indicates the adatom diffusion path on the void surface. We proposed an equation to describe the competition between the ionic fluxes induced by void-growth stripping and LBL stripping

$$\zeta =\text{log}\left(\int_{C_{\text{Li}/\text{Void}}}J_{\text{void growth}}\mathrm{dl}/\int_{L_{\text{Li}/\text{SE}}}J_{\text{LBL}}\mathrm{dl}\right)$$ (1)

where *J*_void growth_ is the flux via adatom diffusion that leads to void growth, and *J*_LBL_ is the flux via LBL stripping; *C*_Li/Void_ is the length of the Li/void interface (red line), and *L*_Li/SE_ is the length of the Li/SE interface (green line); ζ is the logarithm of their ratio, representing the evolution outcomes such as void growth, shrinkage, or stabilization. Figure 3H shows three typical results of ζ that varies with time at different current densities under a stack pressure of 10 MPa. Specifically, during the stripping process, if ζ > 0, the void grows; if −0.85 < ζ < 0, the void remains approximately stable; if ζ < −0.85, LBL stripping dominates, shrinking the void. Figure 3I and movie S8 present the corresponding three sets of sequential simulation images for void growth, stabilization, and shrinkage, respectively. On the basis of our experimental data, a *i-P* map with *P* from 0 to 20 MPa and *i* from 0 to 40 mA cm^−2^ was generated (Fig. 3J). (Note that the data of Fig. 3B are not included in Fig. 3J due to its different boundary conditions, as discussed in figs. S24 and S25).

This map demonstrates that in the low stripping rate and pressure regions (purple), Li diffusion flux dominates, leading to void growth. It is reasonably understandable that under low-rate stripping, adatom diffusion is energetically favorable and sufficient to sustain the stripping current. However, Li^0^ diffusivity is temperature dependent and has a limit at room temperature. As the current density increases, LBL stripping is initiated and gradually dominates the stripping process due to its high efficiency in mass transport, leading to void shrinkage. Higher pressure also favors the LBL stripping, possibly because the vacancy aggregation at the interface tends to be suppressed at high pressure, preventing the void formation. In most intermediate pressure/rate regions (yellow), the void is restricted in small size under the dynamic balance of the aforementioned competition. (See detailed discussion in section S1.1 of the Supplementary Materials.)

On the basis of the above findings, a possible mechanism that can help to suppress void growth in practical SSBs is hypothesized, in which the role of stack pressure is reevaluated. Because of the inhomogeneity of Li metal anodes with polycrystalline nature, only the Li grains (or domains) in intimate contact with SEs can dissolve in a LBL pattern. However, the contact regions will quickly become tensile stressed since the resistance from neighboring Li metal can stop the grains from moving toward the SE. Then, vacancies would aggregate at the interface to form voids, like the case in Fig. 1A. If under stack pressure, then the anode should entirely experience a displacement/drift toward SE. Although the applied pressure may not be evenly transmitted to the whole interface (*41*, *42*), it can still substantially increase the compressive-stressed contact area and facilitate the LBL stripping, thus suppressing the void growth. We need to point out that for the contact areas free of (or far from) voids and GBs in Li metal (*43*), compressive stress is not necessary for LBL stripping (due to the different boundary conditions from the model in Fig. 3G), which can occur even under tensile stress at low current density if the anode can drift (see our experiments, simulations, and discussion in figs. S24 and S25). Therefore, the critical role of stack pressure is to produce a continuous drift toward SEs for the Li anode, thus enabling LBL stripping at the contact areas.

In a more complete picture, creep deformation should be considered (see the schematics and discussion in figs. S26 and S27) (*44*–*47*). The plastic deformation can not only replenish the interface and collapse the voids but also homogenize the applied compressive stress (*7*, *48*), thus facilitating the LBL dissolution at more contact areas. Moreover, it is worth noting that for a Li anode composed of different sizes of single-crystal gains, the applied stack pressure may not reach the yield strength of micron-sized or smaller Li grains (*38*, *49*). In these grains, the formed voids at interface are hard to annihilate by plastic deformation. Thanks to the LBL stripping mechanism, these voids may be restricted from growing or even shrink. The remaining small voids at the stripping interface will not cause obvious polarization (Fig. 1A and fig. S28), and they can be refilled during the subsequent plating (see below).

### Galvanostatic cycles of a symmetric SSB

**Fig. 4.**
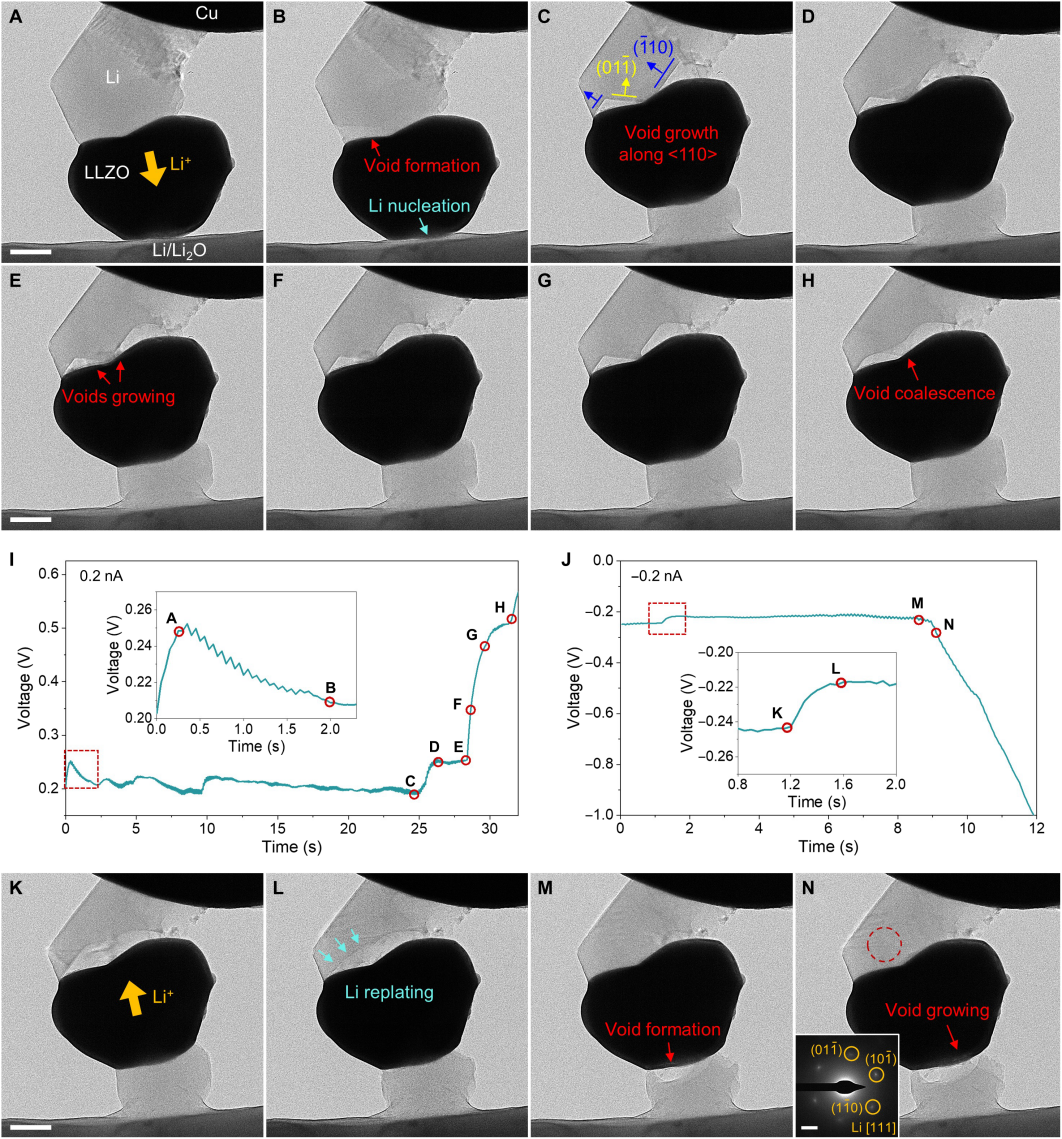
Interfacial evolution of a Li|LLZO|Li symmetric microbattery during a single galvanostatic cycle. (**A** to **H**) Void growth at the upper Li/LLZO interface and Li deposition at the bottom Li/LLZO interfaces upon downward Li transport during the first half cycle. Scale bars, 500 nm. (**I** and **J**) The corresponding *V-t* curves recorded during the first and second half cycles under a constant current of 0.2 nA, respectively. (**K** to **N**) Li replating and void vanishing at the upper interface and void growth at the bottom interface upon upward Li transport during the second half cycle. Scale bar, 500 nm. The inset in (N) is the SAED image of the replated Li taken from the circled area. Scale bar in SAED image, 2 nm^−1^.

In the above stripping experiments, the other (plating) side of LLZO, which formed a large-area contact with the Li substrate, usually had a negligible impact on the total impedance change. However, in a two-electrode symmetric cell, both the anodic and cathodic interfacial evolutions can markedly influence the overall voltage response (*8*, *18*). Thus, a Li|LLZO|Li symmetric SSB containing a micron-sized LLZO particle was in situ constructed (Fig. 4 and movie S9), which enables real-time visualization of both interfaces and the precise correlation of void evolutions to electrochemistry. First, a constant current of 0.2 nA was applied to drive the predeposited Li metal (between LLZO and Cu CC) to dissolve at its interface with LLZO; meanwhile, Li started to nucleate at the bottom of LLZO, i.e., its interface with the Li substrate (Fig. 4B). Accordingly, an appreciable overpotential was synchronously recorded in the voltage curve (Fig. 4I and the inset), which corresponds to the incubation and nucleation of Li metal at the lower Li/LLZO interface (more investigations of Li nucleation will be presented elsewhere). Later, the Li metal nucleus continued to grow and lifted up the LLZO particle (Fig. 4C). The rise of the LLZO particle slowed down the growth rate of the voids at the upper Li/LLZO interface. In this period (2 to 24 s), the voltage underwent small fluctuations, suggesting the competition of the two opposite processes, i.e., the contact loss at upper interface and the contact improvement at lower interface. The exposed surfaces of the voids were identified as the low-energy {110} planes of Li, as revealed by the selected-area electron diffraction (SAED) pattern (the inset in Fig. 4N), exhibiting the tendency to minimize the system energy during void formation. As the lifting of the LLZO was substantially decelerated (Fig. 4D), the dissolution side should experience a notable contact loss, as indicated by a notable rise in the voltage curve (between points C and D in Fig. 4I). Subsequently, the two voids kept growing and gradually merged into a large one (Fig. 4, E to H), leading to a steep rise in the polarization voltage (the contact area at the deposition side remained almost unchanged).

Next, we changed the Li transport direction by applying a reverse current of 0.2 nA. It can be seen that at the upper Li/LLZO interface, Li started to deposit from the TPB on the left side of the void (Fig. 4, K to L). The reduced Li atoms at the TPB tend to diffuse along the fresh surface of parent Li metal, as evidenced by the downward motion of the Li/void interface (indicated by the arrows in Fig. 4L), suggesting an epitaxial-like growth. This is supported by the SAED pattern in Fig. 4N, which shows that the replated Li had the same crystal orientation with the parent Li metal. Moreover, no nucleation overpotential was observed at the beginning of plating, further supporting the epitaxial-like growth model, which is energetically more favorable than Li deposition on a heterogeneous surface (e.g., Li_2_O or LLZO) (*27*). As the void was gradually filled, the recovery of the Li/LLZO contact led to a decrease of the overall voltage (Fig. 4J and the inset). Note that in Fig. 4L, the contact loss at the lower Li/LLZO interface was still limited. However, as the Li kept dissolving at the lower Li/LLZO interface, a void formed (Fig. 4M) and expanded (Fig. 4N), which soon dominated the voltage response, resulting in the severe polarization in Fig. 4J.

**Fig. 5.**
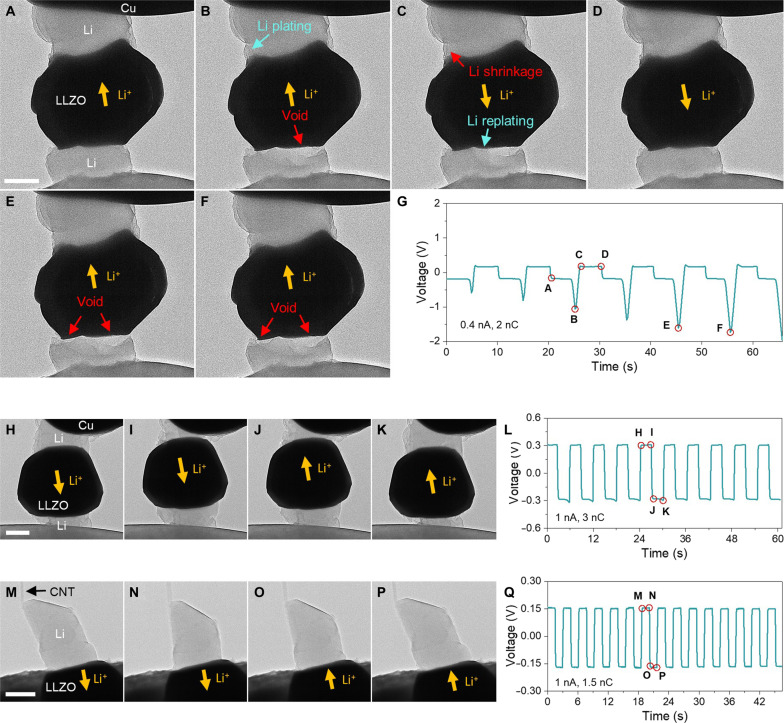
Galvanostatic cycles of Li|LLZO|Li microscale SSBs. (**A** to **G**) Time-lapsed TEM images of a SSB during one of the repeated cycles [(A) to (D)] and in the subsequent two cycles [(E) and (F)], corresponding to the points [(A) to (F)] in the voltage curve at 0.4 nA in (G), where the polarization peaks appear on only one side and become intense because of the enlargement of voids with cycle. (**H** to **L**) TEM images of another SSB during one of the 10 repeated cycles at 1 nA, corresponding to the points in the voltage curve in (L). (**M** to **Q**) TEM snapshots of a SSB with CNT as CC in 1 of the 15 cycles at 1 nA, corresponding to the points in the voltage curve in (Q). No obvious polarization is found in (L) and (Q) due to the void-free stripping. Scale bars, 500 nm.

After the detailed analysis of the voltage response to interface evolution in a single cycle of the SSB, we demonstrated the repeated cycling of our miniature SSBs. Figure 5 (A to D) shows the structural evolution of a symmetric battery in one of the repeated cycles at 0.4 nA. Different from the case in Fig. 4, the voltage curve in 6 cycles shows prominent polarization peaks on only one side, corresponding to the void formation at the lower Li/LLZO interface (Fig. 5B and movie S10). When the void was refilled under the reversed current, the contact area of the upper Li/LLZO interface remains almost unchanged (Fig. 5D). Here, the upper Li metal underwent the expansion or shrinkage at the side wall via adatom diffusion (without changing the position of TPB) upon cycling [indicated by the arrows in Fig. 5 (B and C)]. As a result, the voltage curve remained basically flat on the other side. Notably, the voltage polarization became more severe with the cycling, which can be attributed to the gradual increase of contact loss at the lower interface, because the void filling was not completely reversible upon cycling (Fig. 5, E and F). In addition to the cycling with one-side polarization, we also observed the polarization at both sides of the cyclic voltage curve, where the voids formed at the two Li/LLZO interfaces (fig. S29 and movie S14). Similar polarization features were frequently observed in bulk SSBs (see, e.g., fig. S30), and our results provide a direct microscopic insight, which unambiguously links the cell polarization to the void evolution at Li/SE interfaces.

To avoid voltage polarization, we then explored the cycling based on void-free stripping. As discussed above, this homogeneous dissolution requires the relative movement between Li metal and the SE, which, however, can be hindered by the mechanical resistance from a fixed CC or Li_2_O shells. In the cases of Figs. 4 and 5 (A to G), the voids formation actually originated from the Li_2_O shells formed during the relatively low-rate cycling (0.2 to 0.4 nA). Therefore, we demonstrated two ways to achieve stable Li battery cycling at a higher current of 1 nA. The high-rate cycle can reduce the exposure time of the freshly deposited Li to vacuum and minimize its surface oxidization before the Li was stripped in each cycle. As shown in Fig. 5 (H to L) and movie S11, with the same symmetric cell configuration, the middle LLZO particle can float up and down freely to maintain the Li/SE contact at both interfaces. At the cathodic interface, Li was plated also in a LBL pattern, pushing the LLZO particle toward the anodic side that underwent LBL stripping (Fig. 5, H to K). Consequently, this cell can stably cycle for more than 10 times, exhibiting a symmetric voltage profile without obvious polarization (Fig. 5L).

Also, inspired by the free retraction of Li enabled a CNT as the CC, we conducted a high-rate cycling based on such a cell configuration. The snapshots captured from one cycle shows that the Li metal was repeatedly retracted to and pushed out from the LLZO during cycling (Fig. 5, M to P, and movie S12). This void-free cycling enabled flat voltage trace in each half cycle, resulting in a regular operation of the battery for at least 15 cycles (Fig. 5Q). These results may benefit the design of pressure-free SSBs desired for practical use (*50*, *51*). Such small SSBs enabling stable cycles have never been reported before, which could also inspire the future microbatteries used for powering an autonomous micro/nano device or system (*52*).

## DISCUSSION

In summary, the interfacial evolutions of SSBs under galvanostatic cycling are in situ observed in TEM, linking clearly individual voids to electrochemistry. Two typical Li stripping modes, i.e., void-growth and void-free stripping, are found and precisely correlated to their synchronous voltage responses. On the basis of these two microscopic processes and their competition, we proposed a *i-P* map to describe the void evolution at the Li/SE interface. In particular, a mechanism of void suppression based on LBL stripping is suggested, which differs from and can be an important supplement to the mainstream view based on the creep deformation of Li metal under stack pressure. In addition, the preferential formation of voids at the GB and contamination layers on the LLZO surface are directly visualized. We also demonstrated the cycling of a Li|LLZO|Li symmetric battery, and the microscopic processes that can deteriorate or improve the two interfaces are well correlated with the electrochemistry. In particular, the repeated void-free cycling can be achieved on the microscale batteries by enabling the homogeneous dissolution. The developed TEM characterization method based on in situ constructed micron-sized SSBs cycled in galvanostatic mode can be used to quantitatively study various electrode/SE interface systems (such as sulfide electrolyte–based and Si anode–based SSBs, which will be presented elsewhere). This work provides a universal approach and valuable insights for understanding the void evolution and other interface issues, together with inspiring attempts for designing stable Li/SE interfaces and miniature SSBs.

## MATERIALS AND METHODS

### Synthesis of LLZO

The cubic garnet-type SE LLZO was synthesized using a solid-state synthesis technique. Briefly, LiOH·H_2_O (95%, Aladdin), La_2_O_3_ (99.99%, Aladdin), ZrO_2_ (99.99%, Aladdin), and Ta_2_O_5_ (99.8%, Aladdin) were weighted in stoichiometric ratio and extra 15 wt % of LiOH·H_2_O to compensate for Li loss. The powders were ball-milled with isopropanol as a dispersant at 300 rpm for 12 hours and dried to obtain the precursors. The mixture was then calcined at 900°C for 12 hours in alumina crucible to obtain the pure phase material.

### In situ TEM characterization

The in situ TEM experiment was performed in a FEI Talos-F200s TEM at an accelerating voltage of 200 kV. The beam dose was controlled between 0.16 and 2.5 e·Å^−2^ s^−1^ for in situ imaging to minimize the influence of electron beam. The TEM–scanning tunneling microscopy holder was capable of piezo-driven manipulation and connected to a high-precision current source. In a glove box filled with Ar gas, a Cu rod attached with Li metal was pressed into the LLZO powder dispersed on a glass substrate. The attached LLZO particles can be submerged in the Li metal. Subsequently, the Cu rod was mounted on the movable part of the holder. On the other end of the holder, a Cu tip (sometimes attached with CNTs) or an AFM tip was mounted as a working electrode. The holder was then transferred into the TEM, during which a thin oxidation layer was formed on the Li metal surface due to the exposure to air. Through in situ manipulation, a LLZO particle was brought into contact with the working electrode, and an anode-free miniature SSB was assembled. Then, a constant current was applied to the Li metal counter electrode to drive the Li deposition or dissolution process.

## Supplementary Materials

### The PDF file includes:

Supplementary Text Figs. S1 to S30 Legends for movies S1 to S14 References
