# Supplementary material for: Galvanostatic cycling of a micron-sized solid-state battery: Visually linking void evolution to electrochemistry
Source: Sci Adv. 2025 Apr 4;11(14):eadt4666. doi: 10.1126/sciadv.adt4666 (PMC11970463; doi:10.1126/sciadv.adt4666)
Supplement: Supplementary file 1 — Supplementary Text Figs. S1 to S30 Legends for movies S1 to S14 References [file sciadv.adt4666_sm.pdf]

Science Advances
AAAS

Supplementary Materials for

Galvanostatic cycling of a micron-sized solid-state battery: Visually linking void evolution to electrochemistry

Haowen Gao *et al.*

Corresponding author: Ming-Sheng Wang, mswang@xmu.edu.cn

Sci. Adv. **11**, eadt4666 (2025)
DOI: 10.1126/sciadv.adt4666

The PDF file includes:

Supplementary Text
Figs. S1 to S30
Legends for movies S1 to S14
References

Other Supplementary Material for this manuscript includes the following:

Movies S1 to S14

Supplementary Text

1. Detail of Phase field simulation

1.1 Void evolution mechanism under different constraints

Figure. S1a illustrates the scenario of Li stripping under the fixed current collector (CC), which impedes the drift of Li metal due to the adhesion between Li metal and CC. Therefore, vacancies will generate at the Li/LLZO interface and then accumulate, resulting in void formation. And the vacancy generation also induces a lattice distortion, resulting in a continuous change in lattice parameter referred as stoichiometric strain (*53*). Such a stoichiometric strain leads to the tensile stress in Li metal. Thus, a tension is experimentally measured for the case of Li stripping under the fixed CC (see fig. S24). And our experimental results also suggest that such tension can accelerate the void nucleation and growth.

Conversely, for the case of the pre-existing void under stack pressure, our experimental results suggest that stack pressure can help to suppress the voids. The stress effect on void evolution could be attributed to three possible mechanisms. (1) The charge transfer process of Li dissolution at the Li/LLZO interface could be affected by stress. However, no significant change in voltage under the stress is observed in experiment, indicating that such effect could be ignored. (2) The diffusion of vacancies could be affected by the stress, which could also be ignored because the change of diffusion might require a GPa-level stress (*54*). (3) The void nucleation is affected by the stress. According to Hull D. *et al.* (*55*), at the atomic scale, the vacancies aggregation must overcome an energy barrier that is strongly dependent on stresses. The energy barrier is decreased under tensile stress that promotes the vacancies aggregation and void growth. Conversely, when stack pressure is imposed, vacancies aggregation and void growth would be suppressed by the compressive stress. Therefore, the third mechanism is probably the reason for the suppressing effect of stack pressure on the pre-existing voids.

A pre-existing void can be stabilized at a certain size, neither enlarging nor shrinking in size, especially in intermediate pressure/rate regions. In this situation, if the void enlarges, the remaining contact area will bear an increased compressive stress (P) and current density (i) due to the reduced contact area, which can promote the following layer-by-layer stripping. Then, the void will shrink in size, and the contact area is therefore increased, leading the P/i to decrease, which in return slows down the layer-by-layer stripping. Thus, a dynamic balance is reached between the two opposite processes, i.e. void growth (via adatom diffusion and TPB extension) and void diminishing (via layer-by-layer dissolution).

Consequently, the competition of multiple factors, including the formation, diffusion, and aggregation of vacancies and the effect of stress and current density, leads to three distinct modes of void evolution, namely, void growth, stabilization, and shrinkage (fig. S1b).

In order to quantitatively describe the void evolutions under the multifactorial interaction of the stripping process, a mechanical-electrochemical coupling multi-phase field model is proposed. Different from previous models (*41, 47, 56*), the present model involves the mechanism of formation, diffusion and aggregation of vacancies; the relationship between aggregation of vacancies and the growth of void is established, in which the effect of vacancy concentration and stress on the aggregation is considered; in addition, Butler–Volmer equation is employed to characterize the layer-by-layer dissolution, which not only eliminates vacancies at the interface but also reduces the size of the voids. Based on the model, different Li stripping behaviours in our experiments could be unveiled. Note that the proposed *i-P* map and the experimental data in Fig. 3J may not be precisely suitable to the real bulk cells, but we believe the overall trend predicted by this map can be applied to describe the void evolution of local regions at the Li/SE interfaces.

1.2 Mechanical-electrochemical coupling multi-phase field model for Li stripping

1.2.1 Thermodynamics

To provide a theoretical description of the aforementioned stripping process, we express the Helmholtz free energy, Ψ, of the relevant system, Ω, as follow:

$$\Psi = \int_{\Omega} \psi(\mathbf{p}, \mathbf{c}, \mathbf{d}, \varphi) d\omega \tag{S1}$$

where ψ is the free energy density; $\mathbf{p}$ and $\mathbf{c}$ represent the set of order parameters and concentration variables, respectively; $\mathbf{d}$ is the displacement field; and φ is the electric potential. The concentrations, $\mathbf{c}$, and order parameters $\mathbf{p}$, are further expressed as:

$$\mathbf{c} = \left(c_{\mathrm{Li^+}}, c_{\mathrm{Li}}, c_{\mathrm{e^-}}, c_{\mathrm{Va}}, c_{\mathrm{HLi}} \equiv c_{\mathrm{Li}} + c_{\mathrm{Va}}, c_{\mathrm{Void}}\right) \tag{S2}$$

$$\mathbf{p} = \left(p_1, p_2, p_3\right) = \left(\frac{c_{\mathrm{HLi}}}{c_{\mathrm{HLi}}^{\mathrm{ref}}}, \frac{c_{\mathrm{Void}}}{c_{\mathrm{Void}}^{\mathrm{ref}}}, 1 - p_1 - p_2\right), \text{ respectively.} \tag{S3}$$

In Eq. (S2), the subscript of component of $\mathbf{c}$, 'Li⁺', 'Li', 'e⁻', 'Va', 'Void', 'HLi', represent lithium-ion, lithium atom, electron, vacancy, void and initial host lithium, respectively; $c_{\mathrm{HLi}} \equiv c_{\mathrm{Li}} + c_{\mathrm{Va}}$ means that the sum of the concentrations of lithium atom and vacancy must be a constant and equal to the concentration of initial host lithium; and c_{Void} is the fictitious concentration and its reference value $c_{\mathrm{Void}}^{\mathrm{ref}}$ is equal to $c_{\mathrm{HLi}}^{\mathrm{ref}}$. In Eq. (S3), p_1, p_2, p_3 designate the lithium anode, void and, solid electrolyte (SE), respectively, as shown in fig. S2; c_*/c_*^{ref} ($*$ = HLi or void) represents the dimensionless concentration. Let p_1 and p_2 be dimensionless concentration and $p_3 \equiv 1 - p_1 - p_2$ to ensure that $p_i = 1$ in phase I and p_i varies smoothly to 0 across the boundaries with other phases.

Considering the interfacial, chemical, mechanical and electric contributions, the total Helmholtz free energy density of the system, ψ, can be divided into four parts: $\psi = \psi^{\text{int}} + \psi^{\text{chem}} + \psi^{\text{mech}} + \psi^{\text{elec}}$, where the superscript, 'int', 'chem', 'mech' and 'elec' represent interfacial energy density, chemical potential energy density, mechanical energy density, and electrostatic potential density, respectively. Following Kundin *et al.* (*57*), the interfacial energy density, ψ^{int}, is expressed as:

$$\psi^{\text{int}} = \sum_{\text{i,j}>1}^{n=3}\left(\frac{K_{\text{ij}}}{2}\left|p_{\text{j}}\nabla p_{\text{i}} - p_{\text{i}}\nabla p_{\text{j}}\right|^2 + H_{\text{ij}}p_{\text{i}}^2 p_{\text{j}}^2\right) \tag{S4}$$

where K_{ij} is the scale factor of interfacial energy density for the interface between phases i and j. If a diffused interface of finite thickness, ξ_{ij}, is used to approximate a sharp (zero thickness) interface with interfacial energy (per unit area), ϑ_{ij}, $K_{\text{ij}} = \xi_{\text{ij}}\vartheta_{\text{ij}}$ is obtained (*58*). The second term on the right-hand side is the sum of doublewell energetic functions (*59*) to ensure that stable phases i and j are separated by the energy barrier H_{ij}, which can be related to the interfacial characteristics as $H_{\text{ij}} = W\vartheta_{\text{ij}}/\xi_{\text{ij}}$ where W denotes the barrier height between them. The chemical potential energy density, ψ^{chem}, produces a driving force for the diffusion of reactants and products, expressed as:

$$\psi^{\text{chem}} = \sum_{*}\left(c_* RT\ln\left(\frac{c_*}{c_*^{\text{ref}}}\right) + c_*\left(\mu_*^0 - RT\right)\right)\ \left(* = \text{Li}, \text{Li}^+, \text{Va}\right) \tag{S5}$$

where R, T and μ_*^0 are the ideal gas constant, thermodynamic temperature, and standard chemical potential, respectively. The mechanical energy density is expressed as:

$$\psi^{\text{mech}} = h(p_1)\psi_{\text{Li}}^{\text{mech}} = h(p_1)\left(\frac{1}{2}\left(\left(\boldsymbol{\varepsilon}^{\text{e}}(\mathbf{c},\mathbf{d})\right)^T\cdot\left(\mathbf{D}^{\text{e}}\boldsymbol{\varepsilon}^{\text{e}}(\mathbf{c},\mathbf{d})\right)\right)\right) \tag{S6}$$

where $\mathbf{D}^{\text{e}}$ is the stiffness matrix for the system and $\boldsymbol{\varepsilon}^{\text{e}}$ is the elastic strain tensor. The stiffness matrix is expressed as $\mathbf{D}^{\text{e}} = h(p_1)\mathbf{D}_{\text{Li}}^{\text{e}} + h(p_2)\beta\mathbf{D}_{\text{Void}}^{\text{e}} + h(p_3)\mathbf{D}_{\text{SE}}^{\text{e}}$, where the subscripts 'Li', 'Void' and 'SE' represent the Lithium, void and SE phases, respectively. As the void has no stiffness, a small amount of β is used as the void term coefficient to ensure that the finite element calculation can continue. $h(x)$ is a continuous interpolation function, which is used to deal with material discontinuities between different phases, generally taking the form $h(x) = x^3(10 - 15x + 6x^2)$ (*60*). Under the assumptions of isotropy and small deformation, the elastic strain in the Li-SE system can be expressed as $\boldsymbol{\varepsilon}^{\text{e}} = \boldsymbol{\varepsilon} - \boldsymbol{\varepsilon}^{\text{mis}}$, where $\boldsymbol{\varepsilon}$, $\boldsymbol{\varepsilon}^{\text{mis}}$, are the total strain and stoichiometric strain, respectively. The assumption of small deformation allows the following geometric relation between the total strain, $\boldsymbol{\varepsilon}$, and displacement, $\mathbf{d}$, as $\boldsymbol{\varepsilon} = \{\varepsilon_{\text{ij}}\} = \{0.5(\partial d_{\text{i}}/\partial x_{\text{j}} + \partial d_{\text{j}}/\partial x_{\text{i}}\}$ (i = 1, 2, 3; j = 1, 2, 3). During stripping, the vacancy generation induces a lattice distortion, resulting in a continuous change in lattice parameter referred as stoichiometric strain (*54*), which can be expressed as, $\boldsymbol{\varepsilon}^{\text{mis}} = -\left(c_{\text{Va}}/c_{\text{HLi}}^{\text{ref}}\right)\mathbf{I}$, where $\mathbf{I}$ is the isotropic tensor. The electric potential density, ψ^{elec}, resulting

from the contribution of charged ions (Li^+) and electrons (e^-), is expressed as:

$$\psi^{\text{elec}} = F\left(\varphi_{\text{SE}} c_{\text{Li}^+} - \varphi_{\text{Anode}} c_{\text{e}^-}\right) \tag{S7}$$

where F is the Faraday constant; where φ_{SE}, φ_{Anode} are the electric potential of SEs and anode, respectively.

1.2.2 Reaction kinetics

Following Bazant (*61*), the reaction rate, r, can be expressed as:

$$r = k^0\left(a_{\text{R}} \exp\left(\frac{(1-\rho)\left(\mu_{\text{R}}^{\text{ex}} - \mu_{\text{P}}^{\text{ex}}\right)}{RT}\right) - a_{\text{P}} \exp\left(-\frac{\rho\left(\mu_{\text{R}}^{\text{ex}} - \mu_{\text{P}}^{\text{ex}}\right)}{RT}\right)\right) \tag{S8}$$

where k_0 is the kinetics constant, ρ is the asymmetric parameter. a_{R} and a_{P} are, respectively, the activity of reactants and products.

Considering the reaction $\text{Li} \longrightarrow \text{Li}^+ + \text{Va} + \text{e}^-$, there are $a_{\text{R}} = a_{\text{Li}}$ and $a_{\text{P}} = a_{\text{Li}^+} a_{\text{Va}} a_{\text{e}^-}$, where the a_* is the activity of component $*$. The subscript 'Li^+', 'Li', 'Va', and 'e^-' represent Li^+ in SE, Li in lithium anode, vacancy in lithium anode, and electron, respectively. According to the definition of Bazant (*61*) and the quasi–equilibrium condition of KKS model (*62*), a_* is concentration dependent, given by:

$$a_* = \exp\left(\frac{\partial \psi^{\text{chem}}}{RT \partial c_*}\right) = \exp\left(\frac{\mu_{\text{chem}}^*}{RT}\right) \tag{S9}$$

And $\mu_{\text{R}}^{\text{ex}} - \mu_{\text{P}}^{\text{ex}} = \mu_{\text{Li}}^{\text{ex}} - \left(\mu_{\text{Li}^+}^{\text{ex}} + \mu_{\text{Va}}^{\text{ex}} + \mu_{\text{e}^-}^{\text{ex}}\right)$ is the difference of excess chemical potential between reactant and products. Following Bazant (*61*), the excess chemical potential involves the contributions of electric field and mechanical deformation. It can be defined as follows:

$$\mu_*^{\text{ex}} = \frac{\delta\left(\int_\Omega f - f^{\text{chem}} - f^{\text{int}} dw\right)}{\delta c_*} + \mu_*^0 \tag{S10}$$

In summary, For the reaction: $\text{Li} \longrightarrow \text{Li}^+ + \text{Va} + \text{e}^-$, the reaction rate can be expressed as:

$$r_{\text{a}} = k_{\text{a}}^0 \left(\begin{array}{l} \dfrac{\left(c_{\text{HLi}} - c_{\text{Va}}\right)}{c_{\text{Li}}^{\text{ref}}} \exp\left(\dfrac{(1\text{-}\rho)\left(\mu_{\text{Li}}^0 - \left(\mu_{\text{Li}^+}^0 + \mu_{\text{Va}}^0 - tr(\boldsymbol{\sigma})\big/\left(3c_{\text{HLi}}^{\text{ref}}\right) + F\left(\varphi_{\text{SE}} - \varphi_{\text{Anode}}\right)\right)\right)}{RT}\right) - \\ \dfrac{c_{\text{Li}^+}}{c_{\text{Li}}^{\text{ref}}} \dfrac{c_{\text{Va}}}{c_{\text{Va}}^{\text{ref}}} \exp\left(-\dfrac{\rho\left(\mu_{\text{Li}}^0 - \left(\mu_{\text{Li}^+}^0 + \mu_{\text{Va}}^0 - tr(\boldsymbol{\sigma})\big/\left(3c_{\text{HLi}}^{\text{ref}}\right) + F\left(\varphi_{\text{SE}} - \varphi_{\text{Anode}}\right)\right)\right)}{RT}\right) \end{array} \right) \tag{S11}$$

Eq. (S11) indicates a mechanical–electrochemical coupling during reaction, where $tr(\boldsymbol{\sigma})/\left(3c_{\mathrm{HLi}}^{\mathrm{ref}}\right)$ indicates that the excess chemical potential results from mechanical deformation of the metal electrode. For the void nucleation reaction: Va ⟶ Void, the reaction rate can be found in a similar way:

$$r_{\mathrm{b}} = k_{\mathrm{b}}^{0}\left(\begin{array}{l}\dfrac{c_{\mathrm{Va}}}{c_{\mathrm{Va}}^{\mathrm{ref}}}\exp\left(\dfrac{(1-\rho)\left(\mu_{\mathrm{Va}}^{0}-tr(\boldsymbol{\sigma})/\left(3c_{\mathrm{HLi}}^{\mathrm{ref}}\right)\right)}{RT}\right)- \\ \exp\left(\dfrac{\partial\psi^{\mathrm{int}}}{RT\partial p_2}\dfrac{\partial p_2}{\partial c_{\mathrm{Void}}}\right)\exp\left(-\dfrac{\rho\left(\mu_{\mathrm{Va}}^{0}-tr(\boldsymbol{\sigma})/\left(3c_{\mathrm{HLi}}^{\mathrm{ref}}\right)\right)}{RT}\right)\end{array}\right) \tag{S12}$$

For the layer-by-layer stripping reaction (HLi ⟶ Li^{+} + e^{-}), the reaction rate is expressed as:

$$r_{\mathrm{c}} = k_{\mathrm{c}}^{0}\left(\begin{array}{l}\exp\left(\dfrac{\partial\psi^{\mathrm{int}}}{RT\partial p_1}\dfrac{\partial p_1}{\partial c_{\mathrm{HLi}}}\right)\exp\left(-\dfrac{(1-\rho)\left(\mu_{\mathrm{Li^+}}^{0}+F\left(\varphi_{\mathrm{SE}}^{*}-\varphi_{\mathrm{Anode}}\right)\right)}{RT}\right)- \\ \dfrac{c_{\mathrm{Li^+}}}{c_{\mathrm{Li^+}}^{\mathrm{ref}}}\exp\left(\dfrac{\rho\left(\mu_{\mathrm{Li^+}}^{0}+F\left(\varphi_{\mathrm{SE}}^{*}-\varphi_{\mathrm{Anode}}\right)\right)}{RT}\right)\end{array}\right) \tag{S13}$$

where $\varphi_{\mathrm{SE}}^{*}$ is the voltage at the Li/SE interface away from the void. For the layer-by-layer stripping reaction of the lithium lattice, it represents the rigid body displacement occurring on the surface of the lithium electrode during stripping, so it is controlled by the voltage at the Li/SE interface away from the void, that is, the minimum voltage at the interface.

1.2.3 Governing equation

Since the morphology evolution of lithium is attributed to the void growth and layer by layer stripping, the governing equation of p_1 can be related to the rate equation, r_{b} and r_{c}. According to our previous works (*63*), the rate-type phase field governing equation can be expressed as:

$$\begin{aligned}\frac{\partial p_1}{\partial t} &= L_{\sigma}(K_{12}(p_2\nabla p_1^2 - p_1\nabla p_2^2) + K_{13}(p_3\nabla p_1^2 - p_1\nabla p_3^2) \\ &\quad +2H_{12}p_1p_2(p_1-p_2)+2H_{13}p_1p_3(p_1-p_3)) \\ &-\frac{\partial g_{12}}{\partial p_1}L_{\eta,12}\left(\frac{c_{\mathrm{Va}}}{c_{\mathrm{Va}}^{\mathrm{ref}}}\exp\left(\frac{(1-\rho)\left(\mu_{\mathrm{Va}}^{0}-tr(\boldsymbol{\sigma})/\left(c_{\mathrm{HLi}}^{\mathrm{ref}}3\right)\right)}{RT}\right)-\exp\left(-\frac{\rho\left(\mu_{\mathrm{Va}}^{0}-tr(\boldsymbol{\sigma})/\left(c_{\mathrm{HLi}}^{\mathrm{ref}}3\right)\right)}{RT}\right)\right) \\ &-\frac{\partial g_{13}}{\partial p_1}L_{\eta,13}\left(\exp\left(-\frac{(1-\rho)\left(\mu_{\mathrm{Li^+}}^{0}+F\left(\varphi_{\mathrm{SE}}^{*}-\varphi_{\mathrm{Anode}}\right)\right)}{RT}\right)-\frac{c_{\mathrm{Li^+}}}{c_{\mathrm{Li^+}}^{\mathrm{ref}}}\exp\left(\frac{\rho\left(\mu_{\mathrm{Li^+}}^{0}+F\left(\varphi_{\mathrm{SE}}^{*}-\varphi_{\mathrm{Anode}}\right)\right)}{RT}\right)\right)\end{aligned} \tag{S14}$$

In Eq. (S14), subscripts 12 and 13 represent the Li/Void interface, and the Li/SE interface, respectively. In contrast to Eq. (S11), multiplying $\partial g_{12}/\partial p_1$ by the second term ensures that the reaction occurs only at the Li/Void interface, and likewise multiplying $\partial g_{13}/\partial p_1$ by the third term ensures that the reaction occurs only at the Li/SE interface. Following Kundin *et al.* (*57*), the function g_{ij} is expressed as $g_{ij} = \left(h(p_i)\Big/\sum_{k}^{n} h(p_k)\right)\left(h(p_j)\Big/\sum_{k\neq i}^{n} h(p_k)\right)$.

Similarly, the governing equation of p_2 for void growth can be expressed in form of rate of aggregation, r_b, as follows:

$$\begin{aligned}\frac{\partial p_2}{\partial t} &= L_\sigma(K_{21}(p_1\nabla p_2^2 - p_2\nabla p_1^2) + K_{23}(p_3\nabla p_2^2 - p_2\nabla p_3^2) \\ &\quad +2H_{21}p_2p_1(p_2-p_1) + 2H_{23}p_2p_3(p_2-p_3)) \\ &+\frac{\partial g_{12}}{\partial p_2}L_{\eta,12}\left(\frac{c_{\mathrm{Va}}}{c_{\mathrm{Va}}^{\mathrm{ref}}}\exp\left(\frac{(1-\rho)\left(\mu_{\mathrm{Va}}^0 - tr(\boldsymbol{\sigma})/\left(c_{\mathrm{HLi}}^{\mathrm{ref}}3\right)\right)}{RT}\right) - \exp\left(-\frac{\rho\left(\mu_{\mathrm{Va}}^0 - tr(\boldsymbol{\sigma})/\left(c_{\mathrm{HLi}}^{\mathrm{ref}}3\right)\right)}{RT}\right)\right) \\ &-\frac{\partial g_{23}}{\partial p_2}L_{\eta,23}\left(\exp\left(-\frac{(1-\rho)\left(\mu_{\mathrm{Li^+}}^0 + F\left(\varphi_{\mathrm{SE}}^* - \varphi_{\mathrm{Anode}}\right)\right)}{RT}\right) - \frac{c_{\mathrm{Li^+}}}{c_{\mathrm{Li^+}}^{\mathrm{ref}}}\exp\left(\frac{\rho\left(\mu_{\mathrm{Li^+}}^0 + F\left(\varphi_{\mathrm{SE}}^* - \varphi_{\mathrm{Anode}}\right)\right)}{RT}\right)\right)\end{aligned} \tag{S15}$$

And $p_3 \equiv 1 - p_1 - p_2$.

The Nernst-Planck equation serves to regulate the alterations in the concentration of Li^+:

$$\frac{\partial c_{\mathrm{Li^+}}}{\partial t} = \nabla\left(D_{\mathrm{Li^+}}\nabla c_{\mathrm{Li^+}} + \frac{D_{\mathrm{Li^+}}Fc_{\mathrm{Li^+}}}{RT}\nabla\varphi\right) + r_{\mathrm{Li^+}} \tag{S16}$$

$$r_{\mathrm{Li^+}} = h(p_1, p_3)\left(r_{\mathrm{Li^+}}^{\mathrm{a}} + r_{\mathrm{Li^+}}^{\mathrm{b}}\right) \tag{S17}$$

where $D_{\mathrm{Li^+}} = h(p_1)D_{\mathrm{Li^+}}^{\mathrm{Li}} + h(p_2)D_{\mathrm{Li^+}}^{\mathrm{Void}} + h(p_3)D_{\mathrm{Li^+}}^{\mathrm{SE}}$ is the effective diffusion coefficient of Li^+, and the superscript 'Li', 'Void', and 'SE' represent the lithium anode, void, and solid electrolyte, respectively. In Eq. (S17), $r_{\mathrm{Li^+}}^*$ is a source term provided by the electrochemical reaction for the evolution of Li^+, where $r_{\mathrm{Li^+}}^{\mathrm{a}} = r^{\mathrm{a}}$ represents the rate of lithium dissolution to generate Li^+, and $r_{\mathrm{Li^+}}^{\mathrm{b}} = r^{\mathrm{b}}\left(p_1c_{\mathrm{HLa}}^{\mathrm{ref}} - c_{\mathrm{Va}}\right)/\left(p_1c_{\mathrm{HLa}}^{\mathrm{ref}}\right)$ represents the rate of layer-by-layer stripping to generate Li^+. Similarly, the governing equation for the change of vacancy concentration is as follows:

$$\frac{\partial c_{\mathrm{Va}}}{\partial t} = \nabla\left(D_{\mathrm{Va}}\nabla c_{\mathrm{Va}}\right) + r_{\mathrm{Va}} \tag{S18}$$

$$r_{\mathrm{Va}} = h(p_1, p_3)\left(r_{\mathrm{Va}}^{\mathrm{a}} + r_{\mathrm{Va}}^{\mathrm{b}}\right) + h(p_1, p_2)r_{\mathrm{Va}}^{\mathrm{c}}, \tag{S19}$$

where $D_{\text{Va}} = h(p_1)D_{\text{Va}}^{\text{Li}} + h(p_2)D_{\text{Va}}^{\text{Void}} + h(p_3)D_{\text{Va}}^{\text{SE}} + g_{13}D_{\text{Va}}^{\text{int}} + g_{12}D_{\text{Va}}^{\text{surf}}$ is the effective diffusion coefficient of vacancy, and the superscripts 'int' and 'surf' represent the Li/SE interface and the void surface respectively. In Eq. (S19), r_{Va}^{*} is a source term provided by the electrochemical reaction for the evolution of vacancy, where $r_{\text{Va}}^{\text{a}} = r^{\text{a}}$ represents the vacancy generated by lithium dissolution, $r_{\text{Va}}^{\text{b}} = -r^{\text{b}} c_{\text{Va}} \big/ \left(p_1 c_{\text{HLa}}^{\text{ref}}\right)$ represents the reduction of vacancy in the lattice during the layer-by-layer stripping , and $r_{\text{Va}}^{\text{c}} = -r^{\text{c}}$ represents the vacancy consuming during void nucleation.

The electric field is governed by Poisson's equation:

$$\nabla\left(-\kappa\nabla\varphi\right) = 0 \tag{S20}$$

In Eq. (S20), $\kappa = h(p_1)\kappa_{\text{Li}} + h(p_2)\kappa_{\text{Void}} + h(p_3)\kappa_{\text{SE}}$ is the effect conductivity, where κ_{Li}, κ_{Void}, and κ_{SE} are the conductivity of Li^+ in the lithium anode, void and SE, respectively.

The galvanostatic simulation condition is achieved by introducing the following global constraint during the whole lithium stripping process, only one global equation in Zhang's model (*64*) is considered in this work, taking into account the time required for Li^+ diffusion in the solid electrolyte:

$$I_{\text{ap}} = I_{\text{mt}} \tag{S21}$$

where I_{ap} is the applied current density, which takes positive and negative values during charge and discharge, respectively, and I_{mt} is the current density induced by Li^+ flowing from the far-field boundary. I_{mt} is expressed as:

$$I_{\text{mt}} = \frac{F}{S}\int_S \vec{n}\cdot\left(D_{\text{Li}^+}\nabla c_{\text{Li}^+} + c_{\text{Li}^+}\frac{z_+F}{RT}\nabla\varphi\right)dS \tag{S22}$$

where S is the projected area of the far-field boundary in the vertical direction, z^+ is the valence of cations, and $\vec{n}$ is the outward unit normal vector at the far-field boundary. It notes that, in Eq. (S22), the current density induced by Li^+ transfer is not only affected by the gradient of Li^+ concentration but also the gradient of potential. Thus, the potential at the far-field boundary can be obtained by solving the global constraint (Eq. (S21)).

The displacement field is subject to the equilibrium equation and is expressed as follow:

$$div\left[h(p_1)\mathbf{D}^{\text{e}}\left(\frac{1}{2}\left(\frac{\partial d_{\text{i}}}{\partial x_{\text{j}}} + \frac{\partial d_{\text{j}}}{\partial x_{\text{i}}}\right)\right)\right] = 0 \tag{S23}$$

1.2.4 Void evolution criterion

To clarify the formation mechanism of void, a quantitative analysis and comparison of void growth flux and layer-by-layer stripping flux are introduced. When current density and stack pressure are applied, a large number of vacancies will be generated at the triple-phase-boundary. Most of these vacancies are adsorbed on the surface of pre-existing void and diffuse along the surface of the voids. According to Schmalzried and Janek (*65*), the flux of vacancies injected (annihilated) into the void along the Li/Void interface is defined as the void growth flux, $\boldsymbol{J}_{\text{void growth}}$, as follows:

$$\boldsymbol{J}_{\text{void growth}} = -\left(1 - \frac{c_{\text{Va}}}{c_{\text{Va}}^{\text{ref}}}\right) c_{\text{Va}}^{\text{ref}} \sqrt{\frac{D_{\text{Va}}^{\text{surf}}}{\tau_{\text{Va}}}} \boldsymbol{n}_{\text{Li/Void}} \tag{S24}$$

where τ_{Va} is the relaxation time of the vacancy, and $\tau_{\text{Va}} = c_{\text{Va}}^{\text{ref}} r_{\text{b}}^{-1}$ (*65*), which establishes the relationship between the vacancy relaxation time and the aggregation rate. $\boldsymbol{n}_{\text{Li/Void}}$ is the outward unit normal vector of the Li/Void interface.

The flux of Li^+ migrating away from the interface can be defined as the layer-by-layer stripping flux $\boldsymbol{J}_{\text{LBL}}$. Recent experiments show that the ionic transference number of LLZO is near unity (*66*), and thus it can be assumed that all of the current density in the Li/LLZO interface is generated by the flux of Li^+. With a specific applied current density, $\boldsymbol{J}_{\text{LBL}}$ can be described as:

$$\boldsymbol{J}_{\text{LBL}} = \frac{\boldsymbol{i}_{\text{interface}}}{z_+ F} = \frac{F\left(D_{\text{Li}^+} c_{\text{Li}^+} + c_{\text{Li}^+} \frac{z_+ F}{RT} \nabla \varphi\right)}{z_+ F} \boldsymbol{n}_{\text{Li/SE}} \tag{S25}$$

where $\boldsymbol{i}_{\text{interface}}$ is the interfacial current density and $\boldsymbol{n}_{\text{Li/SE}}$ is the outward unit normal vector of the Li/SE interface.

We then introduce ζ as an evolution mode criterion to indicate whether void formation exists on the interface, and it can be expressed as

$$\zeta = \log\left(\int_{C_{\text{Li/Void}}} \boldsymbol{J}_{\text{void growth}} dl \Big/ \int_{L_{\text{Li/SE}}} \boldsymbol{J}_{\text{LBL}} dl\right) \tag{S26}$$

where $C_{\text{Li/void}}$ is the length of the Li/void interface (red line in Fig. 3G), and $L_{\text{Li/SE}}$ is the length of the Li/SE interface (green line in Fig. 3G). When ζ is less than a small quantity ε_*, which related to

the creep characteristics, geometric scale and other factors of lithium anode, we believe that the void has a tendency to shrink; When ζ is between 0 and ε_*, we consider the void has a tendency to maintain; When ζ is greater than 0, we believe that the void has a tendency to grow, that is:

$$\begin{cases} \text{void shrinkage}, & \zeta < \varepsilon_* \\ \text{void stabilization}, & \varepsilon_* < \zeta < 0 \\ \text{void growth}, & \zeta > 0 \end{cases} \tag{S27}$$

as schematic illustrated in fig. S3.

2. Figures S1 to S30

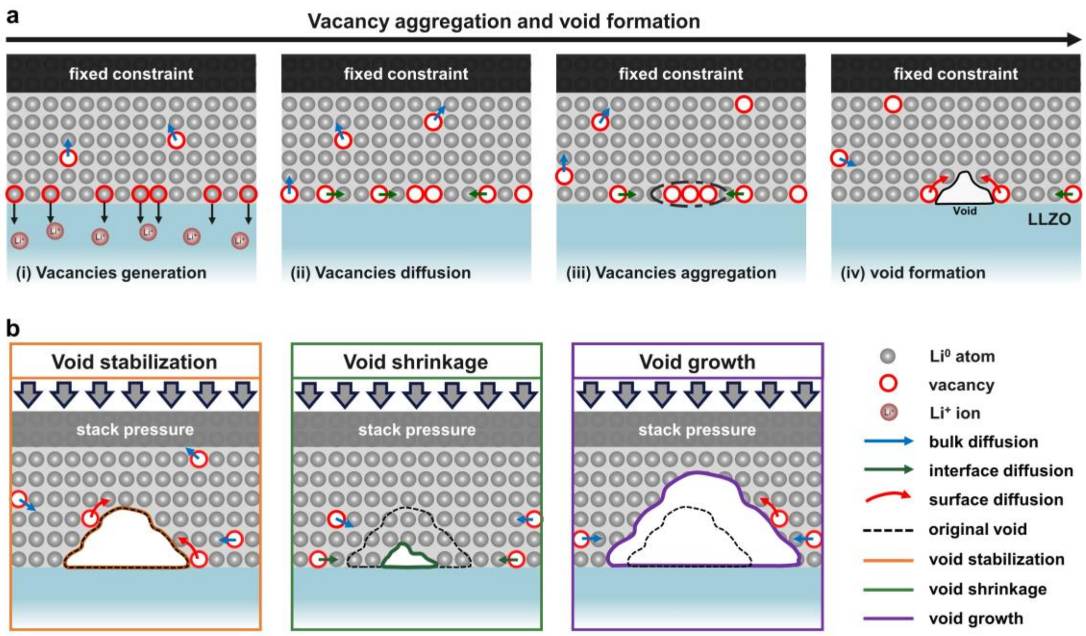

Fig. S1. Schematic illustration of the void formation and evolution. (a) Atomic model illustrating the generation, diffusion and aggregation of vacancies, and void formation during Li stripping with a fixed current collector (CC). (b) Distinct evolution modes of pre-existing void under stack pressure, including void stabilization, void shrinkage, and void growth, respectively, which are caused by the competition of multiple factors.

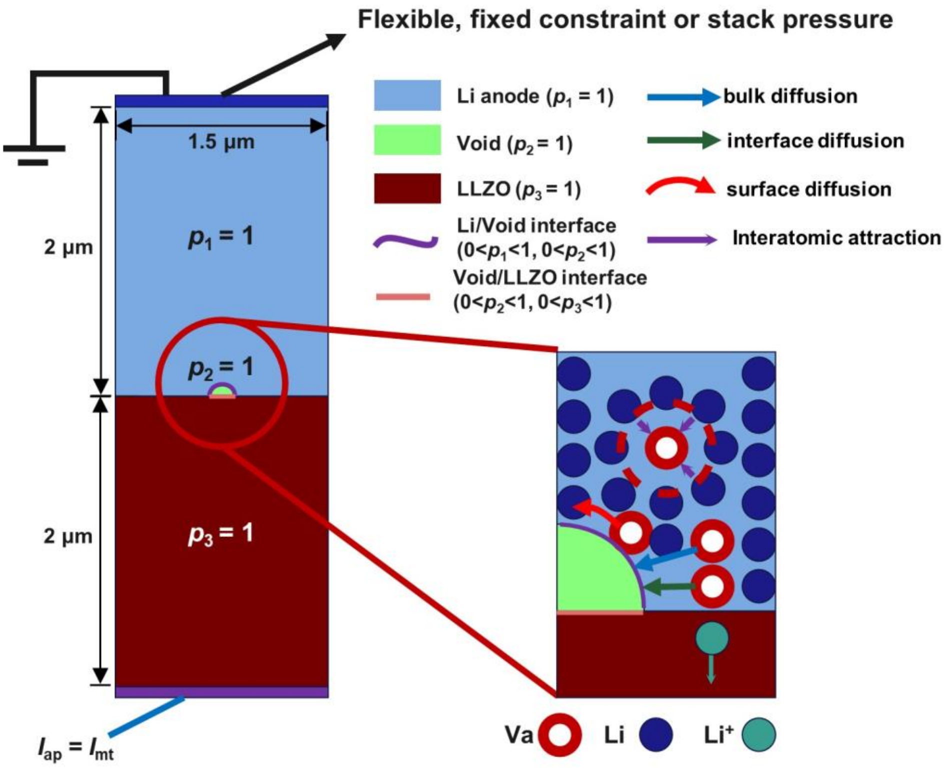

Fig. S2. Geometry and boundary conditions of the model. The boundary condition is set as follows: the surface adatom diffusion on the sidewall of the Li metal is forbidden.

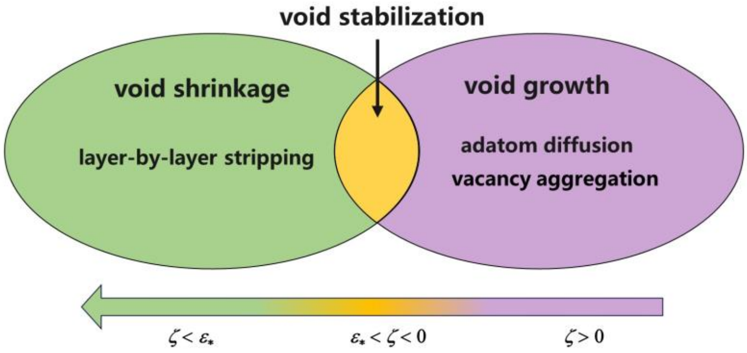

Fig. S3. Criterion to describe the void evolution mode at the interface.

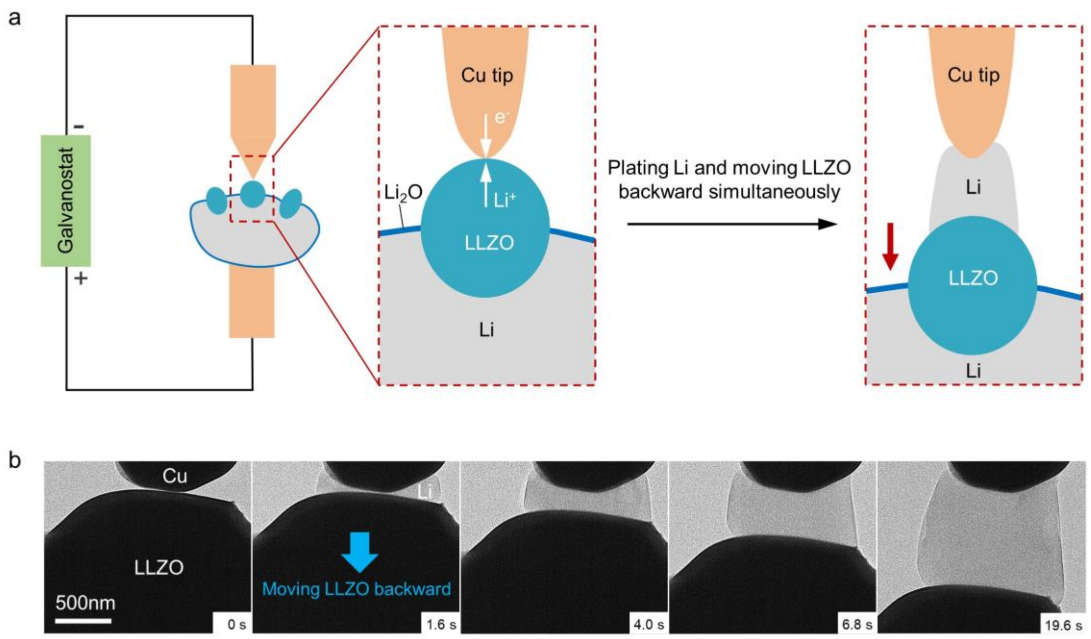

Fig. S4. Construction of a micron-sized anode-free SSB and in-situ Li plating. (a) Schematic of the microscale anode-free solid-state battery setup and in-situ plating Li procedure. The LLZO semi-embedded in Li substrate is manipulated to attach the fixed Cu probe. Then Li is plated between Cu CC and LLZO in a galvanostatic mode, while the LLZO is moved backward simultaneously to increase the gap between Cu and LLZO to accommodate the deposited Li. (b) Time-lapsed TEM images of the in-situ Li deposition process.

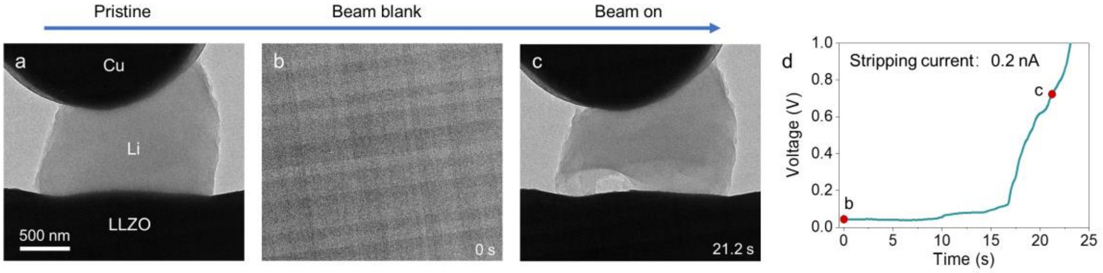

Fig. S5. Li stripping with electron beam blanked off. (a-c) After Li deposition, we turned off the electron beam ('blind' condition). We then applied a constant current of 0.2 nA for some time to strip Li metal. When the polarization voltage increased obviously, we turned on the electron beam to acquire the TEM image, which demonstrated the contact loss between Li and LLZO. (d) Voltage curve recorded during the Li stripping process.

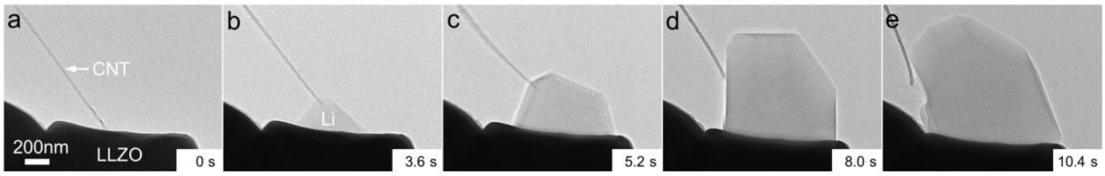

Fig. S6. In-situ plating of a Li particle on the LLZO. (a-e) Time-lapse TEM images of a Li crystal that nucleated at the CNT/LLZO contact point and gradually grew into a faceted particle.

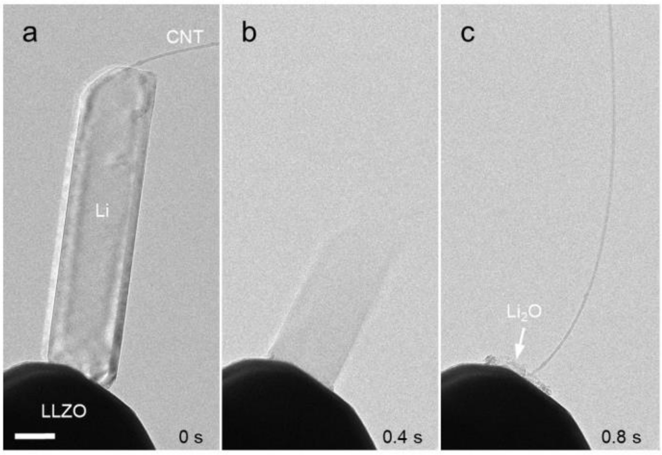

Fig. S7. Li retraction under high-rate stripping induced by a CNT CC. (a-c) Under the galvanostatic loading of 2 nA, the Li whisker was rapidly shortened and fully stripped out, leaving behind Li_2O debris on the LLZO. The stripping current density reached ~2 A·cm^{-2}. Scale bar, 200 nm.

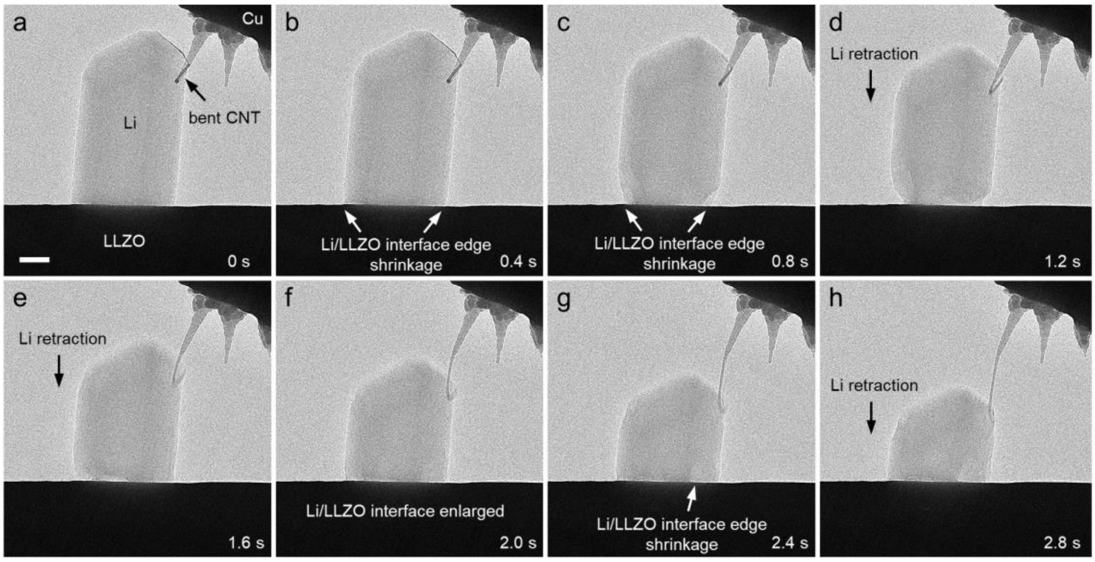

Fig. S8. Reversible switching from TPB dissolution (i.e. interface contraction) to homogeneous dissolution (i.e. retraction) during Li stripping. (a) The fresh Li column with a CNT as CC. (b-c) Under the stripping current of 0.5 nA, surface (adatom) diffusion and TPB dissolution dominated the stripping process, leading to the contraction from the edge of the Li/LLZO interface (i.e. TPB). (d-e) The current density increased with the contraction of the interface area, and Li began to retract towards LLZO. The momentary current density was 108 mA cm^{-2}. (f-g) The homogeneous (layer-by-layer) dissolution in turn caused the contact area to increase, and the current density was reduced to 92 mA cm^{-2}. The interface contracted again, indicating the TPB dissolution. (h) The reduction of interface area again lead to the increase of the current density, initiating the Li retraction once again. Scale bar, 200 nm.

This experiment suggests that with a flexible CC, higher current density favors Li retraction *via* the layer-by-layer dissolution. The threshold current density for triggering the Li retraction in this case was about 100 mA cm^{-2}, but this value would change greatly in different cases, depending on many factors, such as the crystal orientations of the Li metal and LLZO, thickness of the Li_2O layer on the Li crystal and the surface morphology of LLZO, etc.

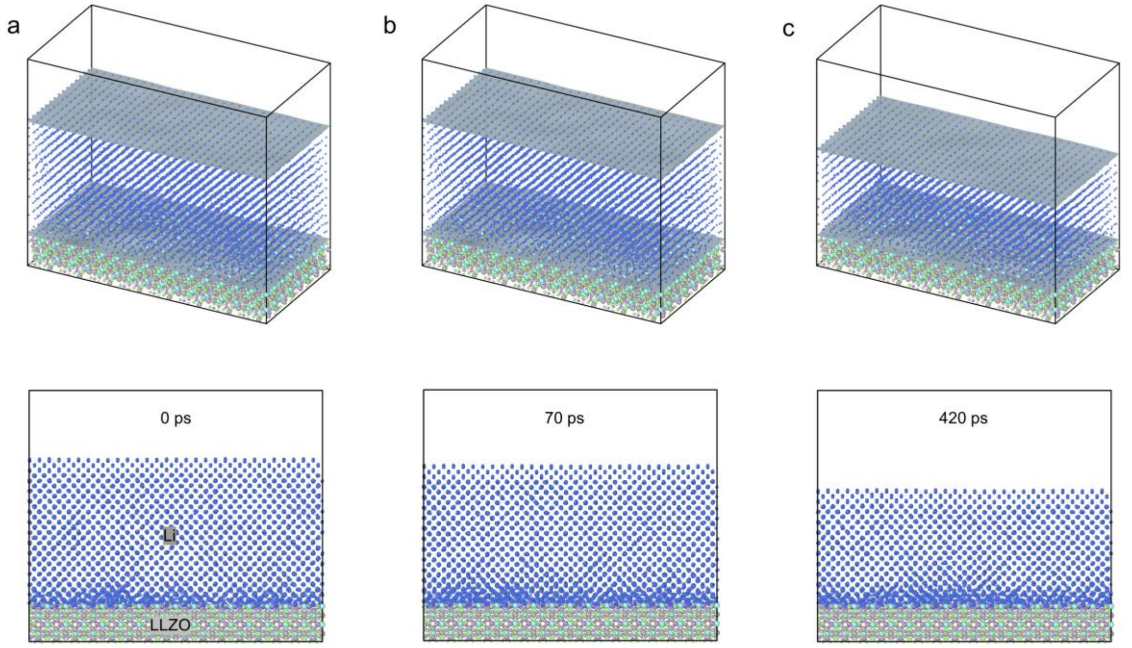

Fig. S9. MD simulation results of homogeneous Li stripping free of constraint. Stereoscopic (above) and cross-section (below) views.

Methods:

Molecular Dynamics (MD) simulations were conducted utilizing the large-scale atomic/molecular massively parallel simulator (LAMMPS) package (*67*). The simulations employed the Li interatomic potential formulated by Nichol et al. (*68*), specifically tailored for Li metal. Interatomic interactions between Li metal and solid electrolytes (SEs) were characterized by a blend of short-range repulsive and long-range attractive forces, commonly implemented in the modeling of the Li metal-LLZO interface (*10, 69, 70*). The short-range repulsive force between metal cation ion i of LLZO and Li atom j of Li metal, separated by a distance r_{ij}, is delineated by:

$$V(r_{ij}) = A_{ij} \exp(-\frac{r_{ij}}{\rho}) \quad (28)$$

The constants A_{ij} and ρ for the interactions involving Li^{+}–Li, La^{3+}–Li, and Zr^{4+}–Li of LLZO in contact with Li metal were sourced from references (*10, 69, 70*). The interaction between the O^{2-} ion and Li atom was modeled using the 8-6 Lennard-Jones (LJ) potential, with parameters derived

from previous studies (*10, 69, 70*). The simulation framework comprised a Li metal slab juxtaposed with a rigid LLZO electrolyte, encompassing 15×30×64 conventional unit cells, each with dimensions of 5.2 nm × 10.4 nm × 5.2 nm. Periodic boundary conditions were applied along the axes perpendicular to the Li–SE interface plane. The system was equilibrated for 50 ps at 300 K to stabilize the Li–SE interface. The Li stripping process was simulated by selectively removing Li atoms situated 5 Å away from the LLZO, while for the layer-by-layer (LBL) stripping process, approximately 5% of the surface Li atoms were removed every 10 ps, with the top surface of the Li metal remaining free. In the void growth simulations, 5% of Li atoms within a cylindrical region of 1.2 nm diameter were removed every 10 ps, alongside a 0.5% removal in other regions, stabilization a fixed top surface. Visualization of atomic configurations was using the Ovito software (*71*).

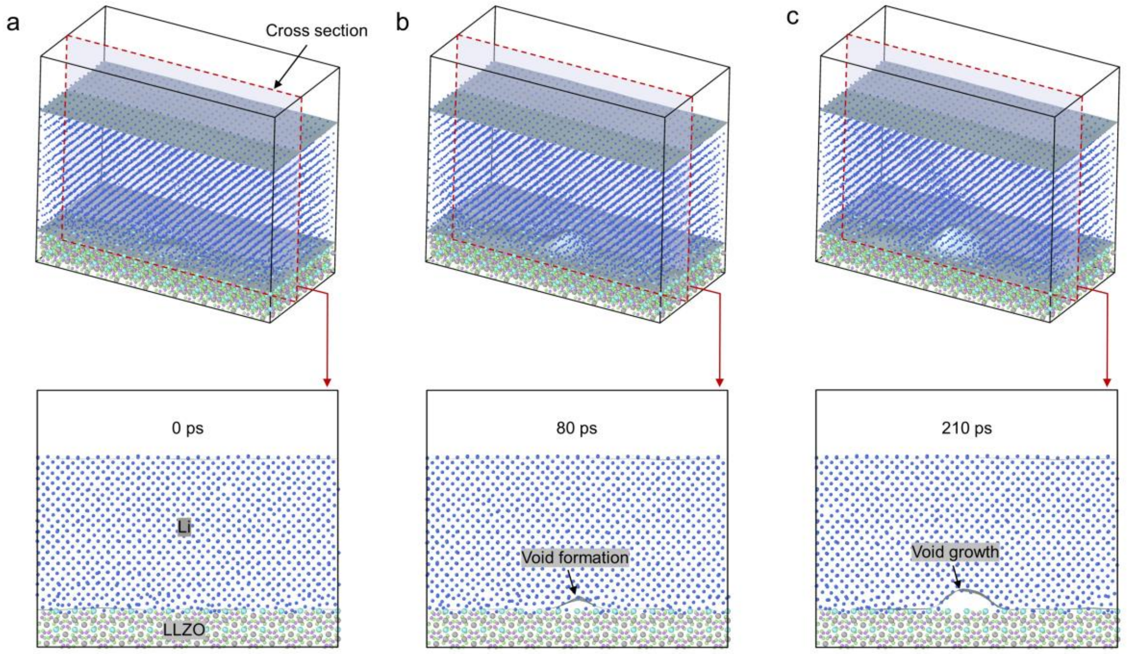

Fig. S10. MD simulation results of void formation under a fixed constraint which impedes the drift of Li metal. Stereoscopic (above) and cross-section (below) views.

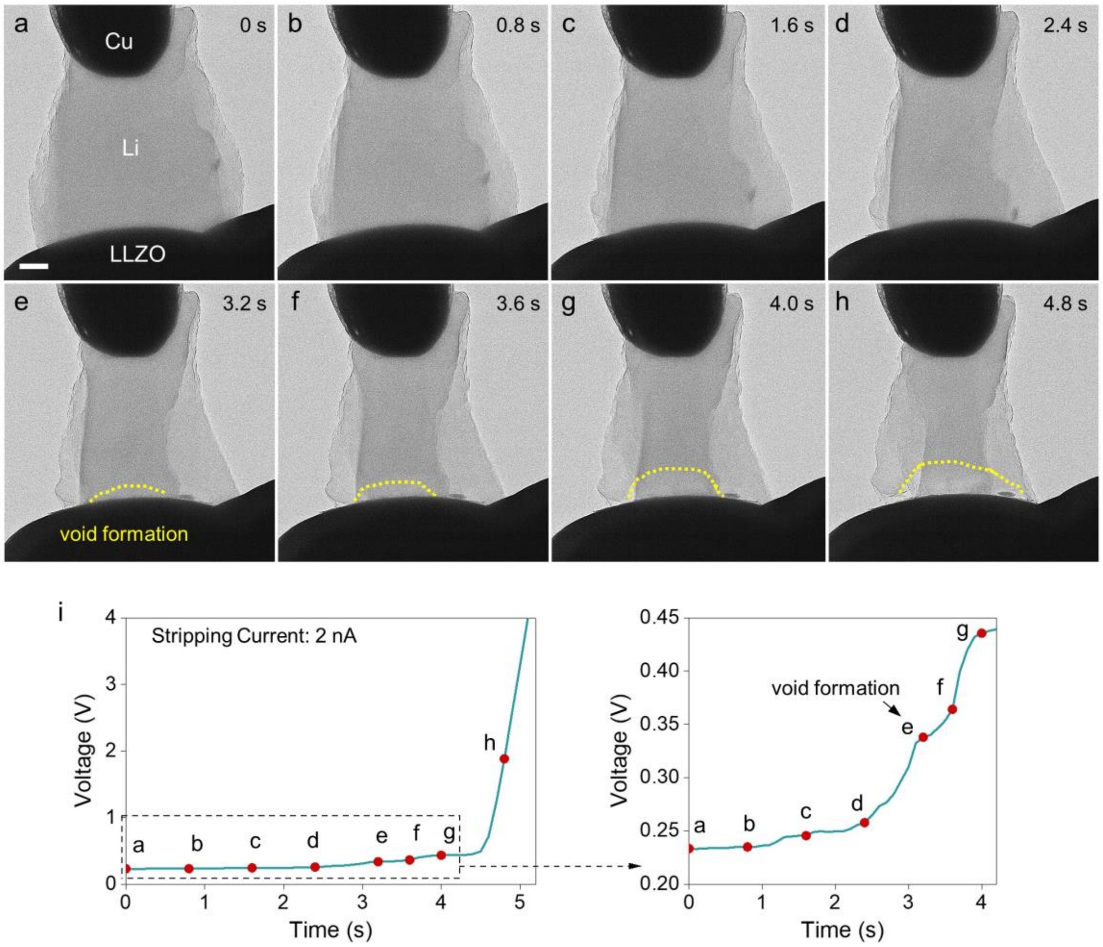

Fig. S11. The adhesion between Li and Cu CC hindering the Li retraction toward LLZO at high stripping rate. (a-d) Due to the adhesion between Li metal and Cu tip, the retraction of Li was hindered. The initial current density was about 120 mA cm^{-2}. To maintain the stripping current, Li metal kept shrinking laterally, resulting in the reduction of the Li/LLZO interface. (e-h) Contact loss between Li and LLZO due to the void formation and enlargement (indicated by the yellow dashed line). (i) Voltage curve recorded during the Li stripping process, suggesting that the area reduction of Li/LLZO interface induced by Li contraction and void formation leads to the increasing polarization voltage. Scale bar, 200 nm.

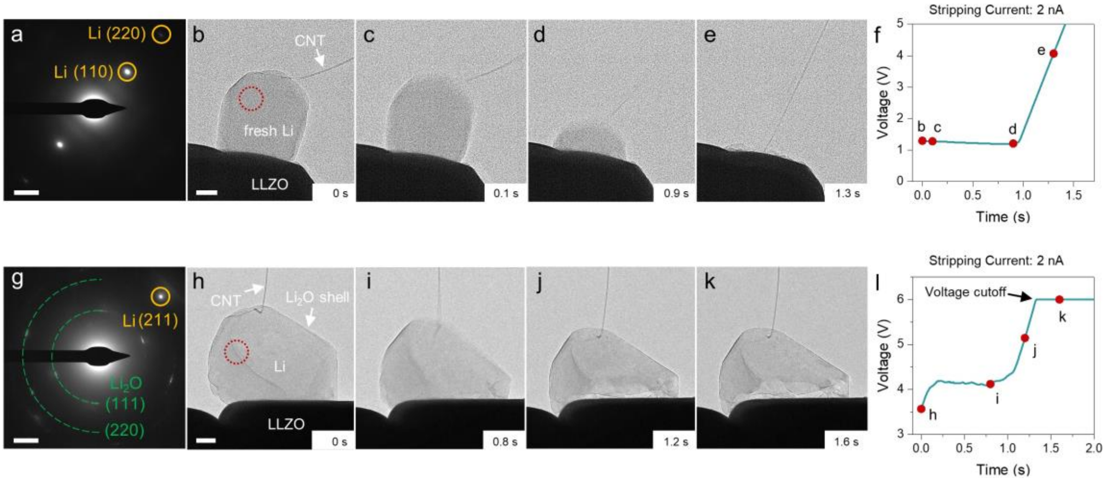

Fig. S12. Effect of Li_2O shell on impeding the retraction of Li at high stripping rate. (a) SAED pattern of the fresh deposited Li particle from the circled area in (b). (b-e) The fresh Li metal without Li_2O shell could be almost completely stripped in 1.3s. Due to the short duration of the high-rate stripping, the deposited Li was barely oxidized into a Li_2O shell, and no voids appeared during the Li metal retraction process. (f) The response voltage curve corresponding to the stripping process in (b-e). (g) SAED pattern of the Li particle with a Li_2O shell from the circled area in (h). (h) The deposited Li was kept in the TEM for several minutes to form a Li_2O shell. (h-k) The voids emerged under the high-rate stripping at 2 nA. (l) The corresponding synchronized voltage trace, which shows a prominent increase in voltage when the Li metal is only partly stripped. Scale bars, (a) 2 nm^{-1}; (b) 200 nm; (g) 2 nm^{-1}; (h) 200 nm.

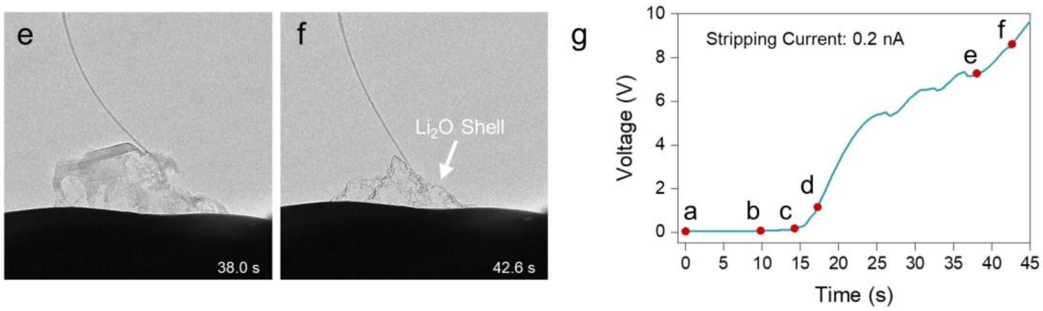

Fig. S13. Low-rate galvanostatic stripping of Li. (a-f) Sequential TEM snapshots of Li stripping at 0.2 nA with a CNT as CC. The current density is ~13 mA cm^{-2}. (g) The corresponding voltage curve. The formed Li_2O shell was clearly seen, and the voids formed at the interface and on the shell. The polarization voltage reached ~10 V before the Li metal was completely stripped, leaving behind the Li_2O debris.

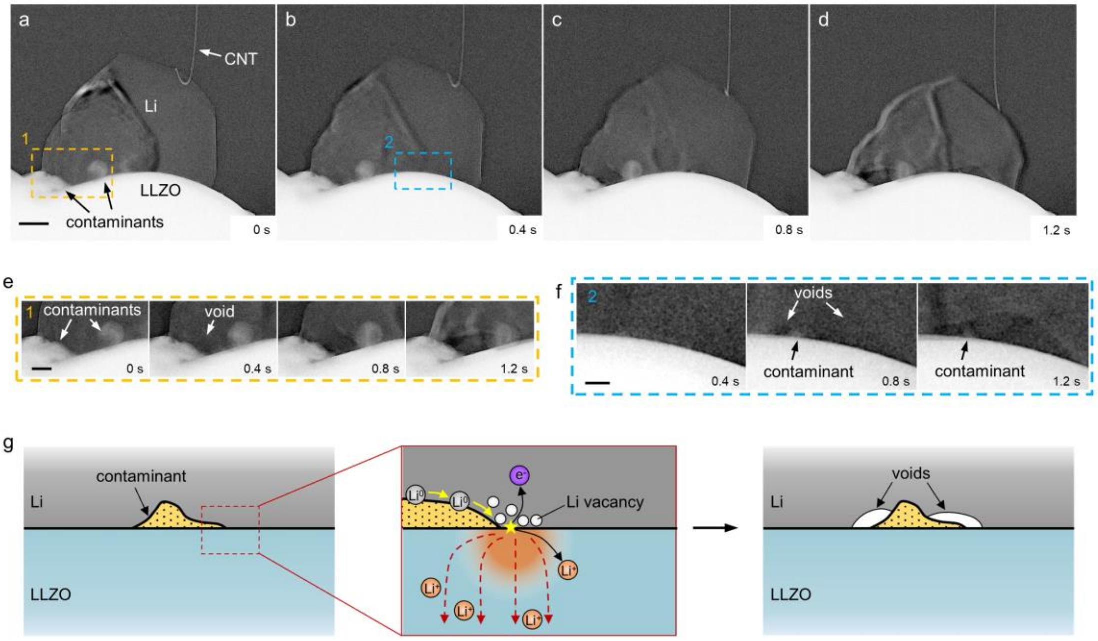

Fig. S14. Void formation at the surface contaminant sites during Li stripping. (a-d) Time-resolved TEM images (contrast-inversed) of the void formation at the edge of contaminants on LLZO. The stripping current is 0.5 nA. (e-f) Magnified TEM images from the boxed areas in (a) and (b), respectively. (g) Schematical illustration of Li stripping on LLZO with surface contamination. Scale bars, (a) 200 nm; (e) 100 nm; (f) 50 nm.

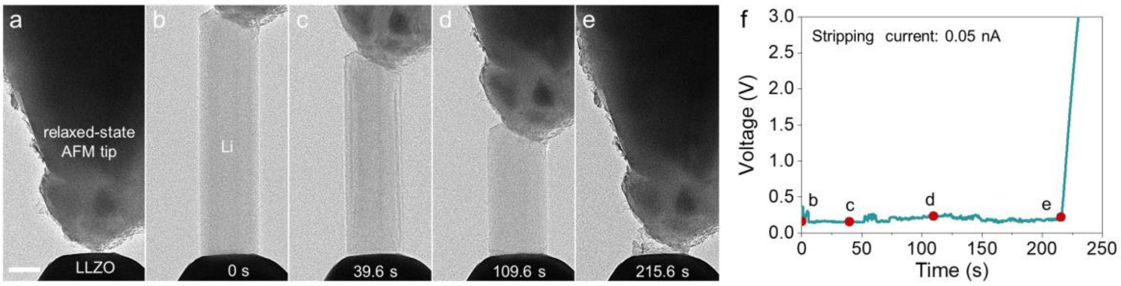

Fig. S15. Li stripping under compressive stress and the corresponding voltage curve. (a-e) The sequential TEM snapshots (k=2.8 N m^{-1}). (f) The corresponding voltage curve. This curve remained roughly flat, ending with an acute increase in polarization upon Li depletion. The slight fluctuations in the curve may originate from the lithiation of the Si AFM tip that changed its conductivity, and also from the mechanical instability of the system under stack pressure. Scale bar, 500 nm.

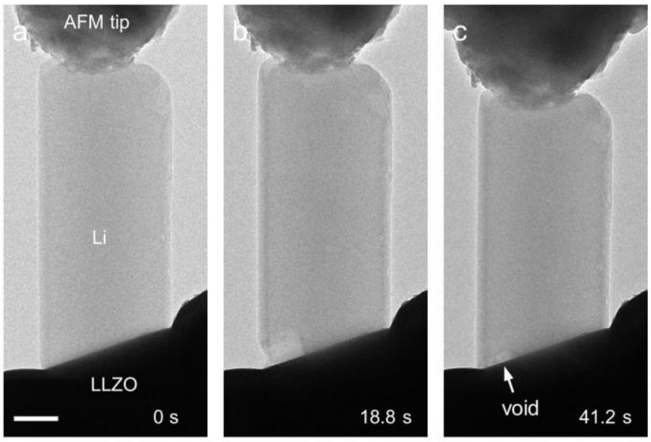

Fig. S16. Li whisker stripping process under stack pressure at a low rate. The initial stack pressure was ~5 MPa and the current density was 3 mA cm^{-2}, respectively (k=2.8 N m^{-1}). Scale bar, 500 nm.

Before investigating void suppression during stripping, we intentionally induced a void at the Li/LLZO interface in a Li whisker. Specifically, after the growth of the Li whisker, the void was intentionally induced under low stripping rate conditions. As shown in fig. S16, at the initial stage of low-rate stripping, the constant current is mainly provided by the Li surface diffusion on the sidewall of the whisker, which leads to the contraction of the Li/LLZO interface, causing the Li metal near the TPB to collapse inward into a cavity. Subsequently, the current density and stack pressure increase with the reduced contact area, and the stripping current contribution of layer-by-layer stripping becomes more significant. As a result, the entire Li whisker drifts toward the LLZO under pressure, and the void shrank in size but was still maintained at the interface.

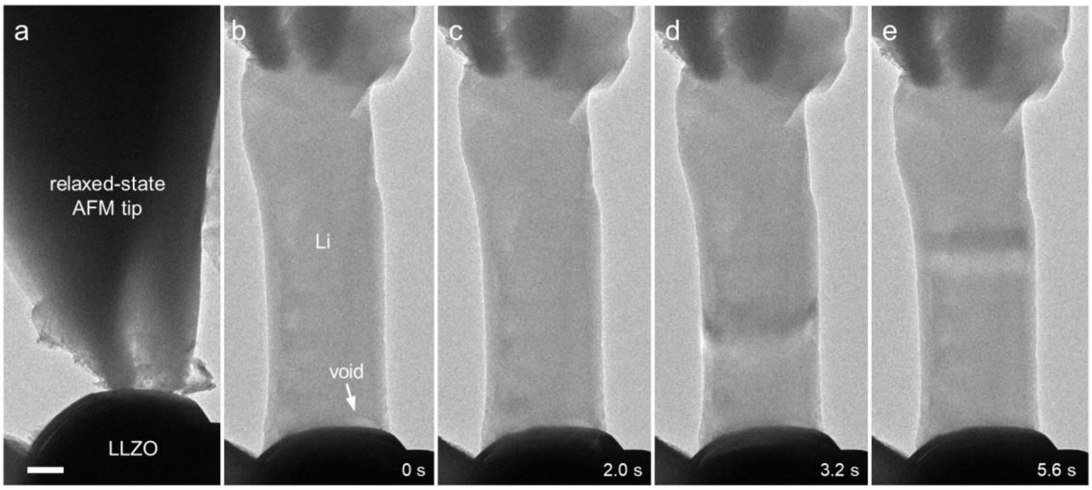

Fig. S17. Sequential TEM images of void elimination under stack pressure with higher stripping rate. The initial stack pressure was ~5 MPa and the current density was 37 mA cm^{-2}, respectively (k=2.8 N m^{-1}). Scale bar, 500 nm.

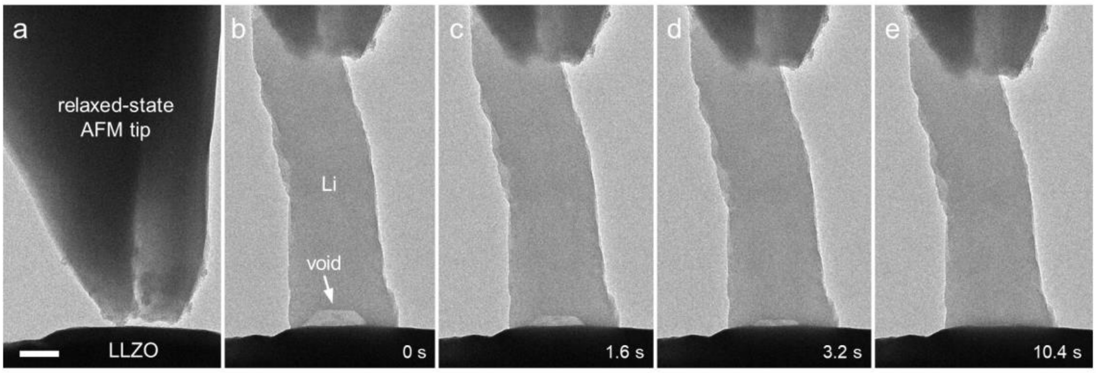

Fig. S18. Sequential TEM images of void elimination under stack pressure with higher stripping rate. The initial stack pressure was ~10 MPa and the current density was 35 mA cm^{-2}, respectively (k=2.8 N m^{-1}). Scale bar, 500 nm.

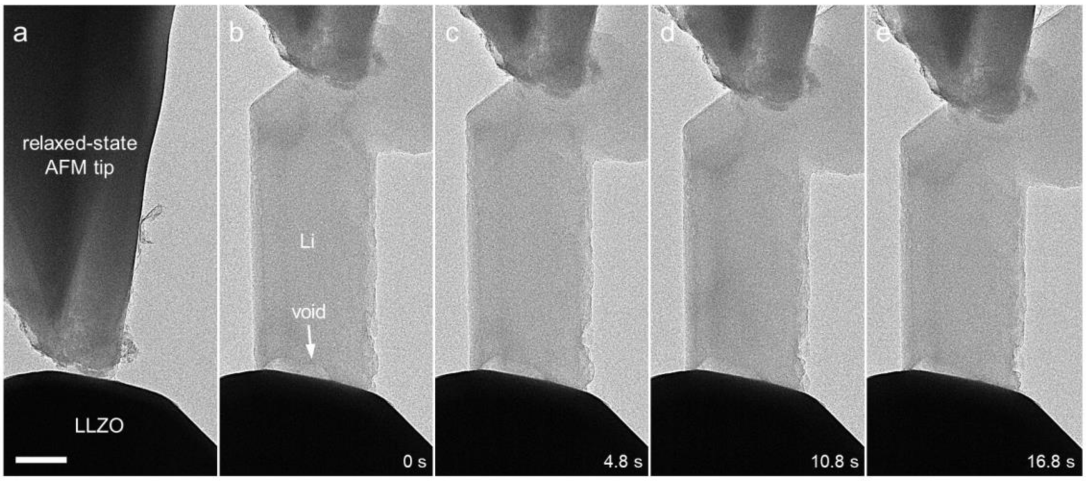

Fig. S19. Sequential TEM images of the void suppression under intermediate pressure/rate conditions. The initial stack pressure was ~11 MPa and current density was 18 mA cm^{-2} (k=2.8 N m^{-1}). Scale bar, 500 nm.

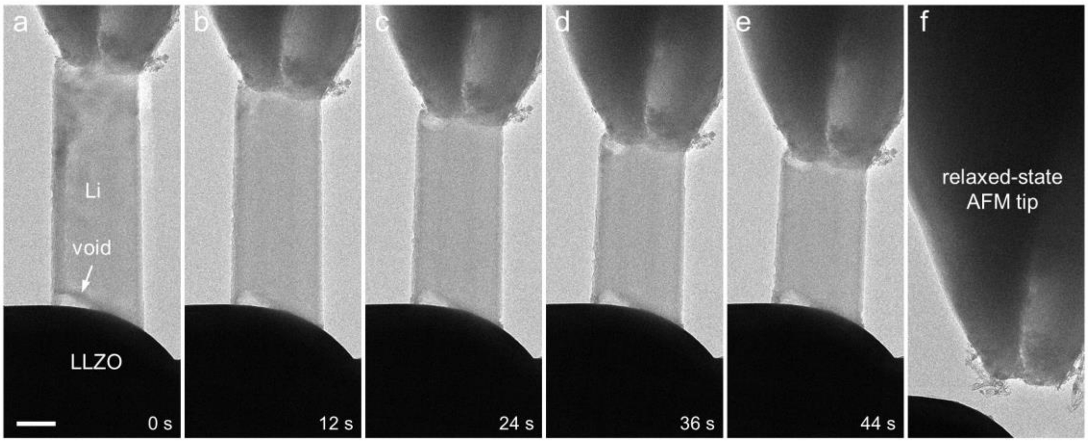

Fig. S20. Sequential TEM images of the void suppression under higher stack pressure. The initial stack pressure was ~15 MPa and current density was 16 mA cm^{-2} (k=2.8 N m^{-1}). Scale bar, 500 nm.

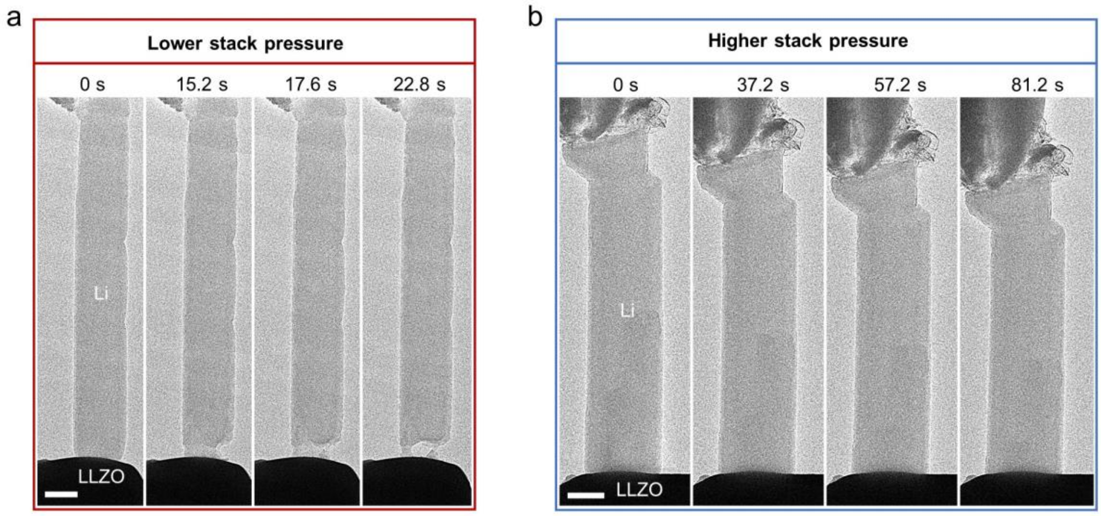

Fig. S21. Effect of stack pressure on Li stripping behaviour. (a) Li whisker stripping under stack pressure of ~2 MPa and current density of 10 mA cm^{-2}. The spring constant of the AFM cantilever is 0.2 N m^{-1}. In order to avoid the influence from Li_2O, after the growth of a Li whisker, we immediately reversed the polarity of constant current to drive Li stripping. The fresh Li at the whisker root shrank *via* surface diffusion and TPB dissolution, resulting in the formation of a cave at the whisker root. In contrast, as shown in (b), the fresh Li at the whisker root was stripped layer-by-layer under higher stack pressure of ~15 MPa at 10 mA cm^{-2}. The spring constant of the AFM cantilever is 2.8 N m^{-1}. Scale bars, 500 nm. This comparison shows that higher pressure favors the homogeneous (void-free) stripping, while the lower pressure favors non-homogeneous (void-forming) dissolution.

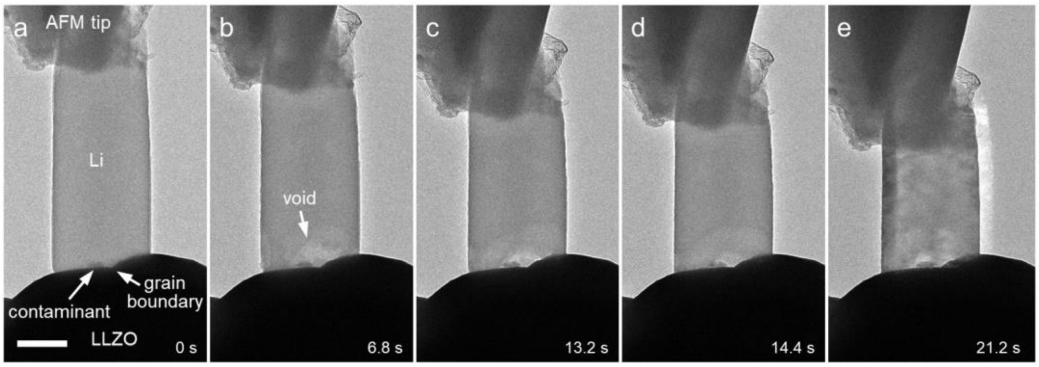

Fig. S22. Void formed at the contaminant and LLZO grain boundary site and restricted in size under stack pressure. (a-e) Sequential TEM snapshots of the Li stripping process under stack pressure, where a void was formed and then restricted in size by layer-by-layer stripping. The initial pressure was ~7 MPa and the current density was ~40 mA cm^{-2} (k=2.8 N m^{-1}). Scale bar, 500 nm.

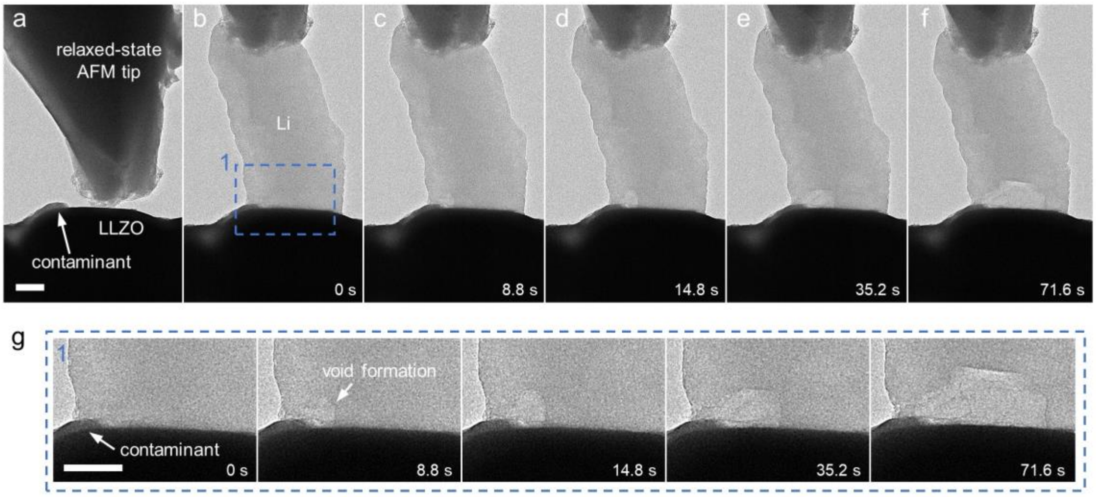

Fig. S23. Void formation and growth at the contamination site under stack pressure. (a) The relaxed-state AFM tip in contact with the LLZO. (b-f) Sequential TEM snapshots of the Li stripping process under stack pressure, where a void was formed and grew around the contaminant. The initial pressure was ~4 MPa and the current density was ~6 mA cm^{-2} (k=2.8 N m^{-1}). (g) The enlarged images at the contamination site from the boxed region in (b). Scale bars, 500 nm.

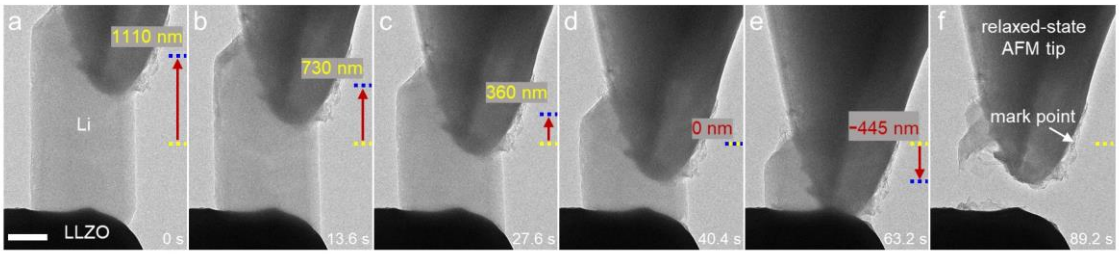

Fig. S24. Homogeneous Li stripping under the load exerted by an AFM probe that transits from compressive stress to tensile stress. (a-f) Sequential TEM images showing the Li whisker stripping process. A relatively higher current density of 25 mA cm^2 was applied, which favors LBL stripping. The stripping of the whisker was initiated under a pressure of ~3 MPa, and the compressive stress decreased with the shortening of Li whisker. As the stress decreased to zero, LBL stripping was still maintained even after the transition to an increasing tensile stress. The maximum tensile deflection of AFM cantilever is ~445 nm, corresponding to a maximum tensile stress of ~1 MPa (k=2.8 N m^{-1}). Scale bar, 500 nm.

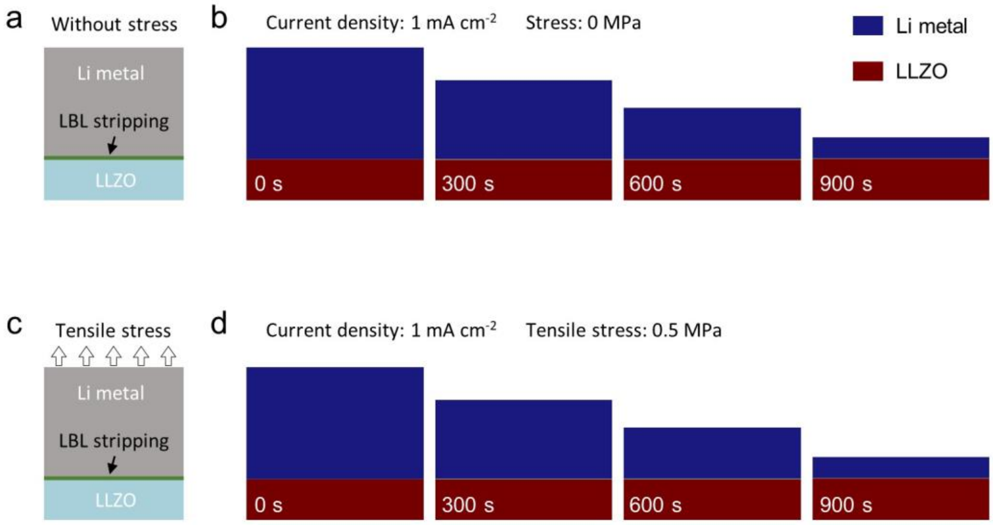

Fig. S25. Phase-field simulation results of homogeneous Li stripping at lower current density under zero stress or tensile stress with a drifting CC. (a) The schematic illustration of the model for phase-field simulation without external stress. To mimic the stripping process in the contact region free of (or far from) void, the boundary condition is set as follows: the surface adatom diffusion on the sidewall of the Li metal is forbidden. (b) Simulation result of LBL stripping under 0 MPa at 1 mA cm^{-2}. (c) The model for phase-field simulation under tensile stress. The boundary condition is the same with that in (a). (d) Simulation result of LBL stripping under tensile stress of 0.5 MPa at 1 mA cm^{-2}. In these two cases, no void formation was found throughout the stripping process, confirming the importance of the drift of Li metal anode on homogeneous stripping.

Discussion:

To mimic the stripping process in the contact region free of (or far from) void, the boundary condition of the phase-field simulation model is set to forbid adatom diffusion on the sidewall of the Li metal. Thus, homogeneous Li stripping will occur if the Li metal anode can drift. Such a boundary condition can also be met in our in-situ TEM experiments. In general, surface diffusion is active on the root surface of Li whisker, TPB dissolution would happen under low current density and stack pressure. However, if a Li_2O layer is formed on the sidewall of deposited Li metal (due to the trace amounts of oxygen in the TEM), which can sometimes inhibit the surface adatom diffusion. Besides, some crystallographic surfaces of Li metal may have a lower Li^0 diffusivity. As a result, as shown in fig. S15 (i.e. Fig. 3B), despite the compressive stress decreasing to near zero, LBL

stripping is maintained throughout the process regardless of the low current density, which is also supported by the above phase-field simulation in fig. S25.

In contrast, in the model of Fig. 3G, the pre-existing void provides the free surface on which adatom diffusion is allowed (but forbidden on the sidewall as well). This is consistent with the experiment of void evolution during stripping, where the void surface stays fresh as the adatoms on the surface continuously diffuse to the TPB dissolution site, preventing Li_2O formation.

For the Li/LLZO interface free of voids, both our experimental and simulation results suggest that LBL stripping can occur even under tensile stress. In this context, the adhesion force between Li metal and LLZO plays a critical role in stabilization the interface contact. However, if the tensile stress continues to increase to reach the limit of the adhesion force between Li and LLZO, voids are expected to form. This is actually the case of Li stripping under fixed constraint (Fig. 1A and fig. S11), where the local tensile stress at the interface can sharply increase upon stripping, so the void has to form at the most tensile-stressed site.

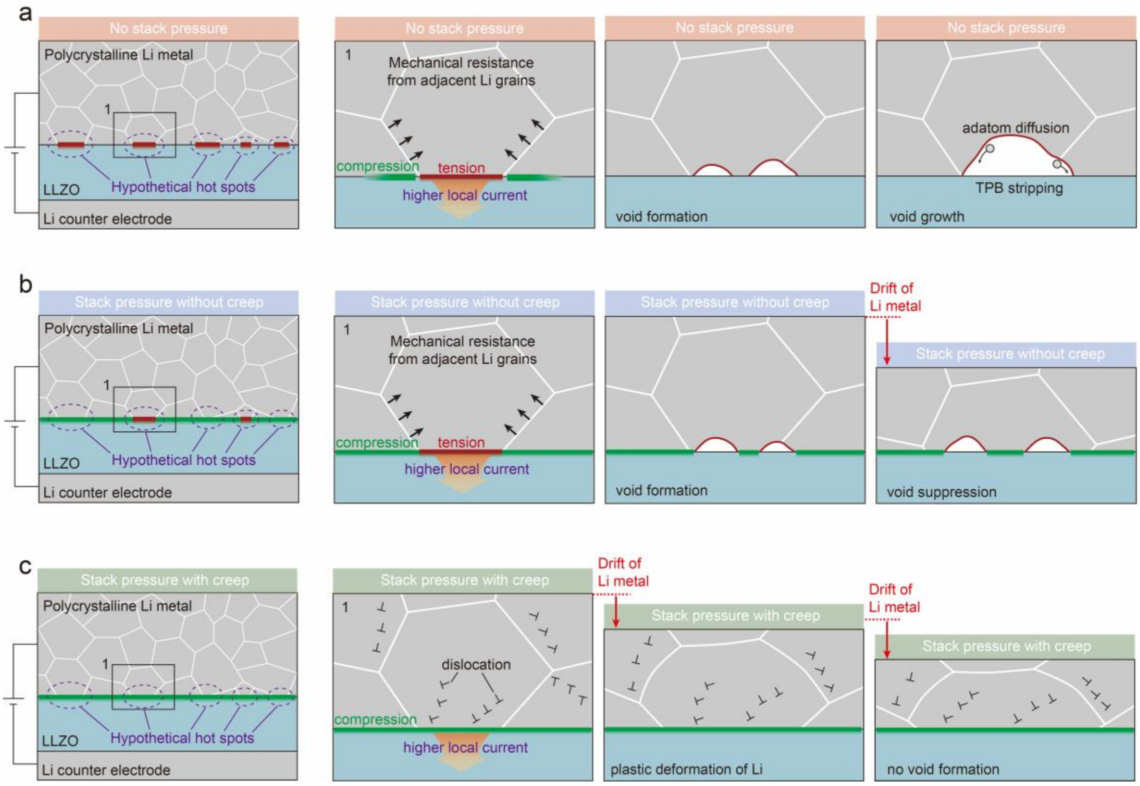

Fig. S26. Schematic illustration of void formation, evolution and suppression in polycrystalline Li metal anode of bulk cell in different scenarios.

In these scenarios, we assume that there are no pre-existing voids at the interface before stripping. (a) In the first scenario without stack pressure, due to the inhomogeneity of the interface between polycrystalline Li metal and SE, there are some hotspots with higher local current density, where LBL stripping is more likely to occur at the beginning of stripping. The Li grain at the hotspot needs to drift to support continuous LBL stripping, which, however, is impeded by adjacent Li grains. Thus, tensile stress builds up at the interface between the Li grain and SE (red line), promoting vacancy generation and aggregation to form voids. Once the void is formed, Li adatom diffusion along the void surface would dominate the stripping current, especially at low current density, leading the voids to grow. (b) In the second scenario, we assume that Li is stripped under stack pressure that does not cause creep deformation. The Li anode has a trend to drift toward SE under the stack pressure. The applied pressure can be transmitted to the interface and increase the compressive-stressed area (green line), but not that uniformly without creep deformation. Thus, voids can still form at the interface area with tensile stress (red line). Thanks to the drift cause by

stack pressure, the remaining contact area experience the LBL stripping, which can suppress void growth from growing larger, as demonstrated in Fig. 3E, and figs. S19 to S20. (c) In the third scenario, Li is stripped under stack pressure with creep deformation. The compressive stress drives the slide or climb of pre-existing or newly generated dislocations in Li grains. The plastic deformation can replenish the interface and largely homogenize the applied compressive stress, which can maximize the compressive-stressed interface area (green line), thus facilitating LBL stripping and further avoiding void formation.

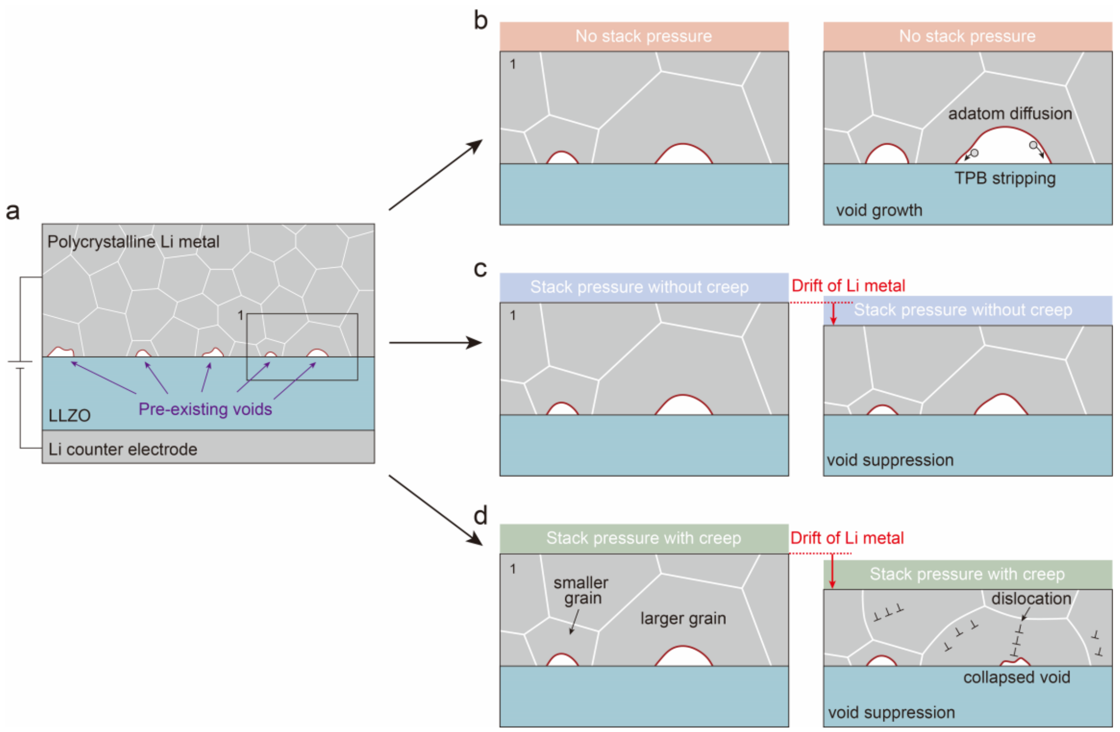

Fig. S27. Schematic illustration of void evolution at the imperfect interface between polycrystalline Li metal anode and LLZO in a bulk cell in different scenarios.

In these scenarios, we assume that there are pre-existing voids at the interface before stripping (a). (b) In the first scenario without stack pressure, the pre-existing voids will grow *via* TPB stripping. (c) In the second scenario, Li is stripped under stack pressure without creep deformation. The compressive stress promote the drift of Li grains, which can suppress the growth of the pre-existing voids. (d) In the third scenario, Li is stripped under stack pressure with creep deformation.

This leads to the plastic deformation of larger Li grains and the collapse of voids, and facilitates pressure transmission and LBL stripping at more contact areas. However, for the smaller Li grains at the interface, plastic deformation is harder to occur to collapse the void inside due to their higher yield strength. Nevertheless, the void growth is inhibited by the LBL stripping enabled by the drift of the grain.

It is noteworthy that LBL stripping **should be a universal phenomenon in real SSBs**, which is possible to occur for all the Li/SE interface with intimate contact. For example, after void collapse by Li creep deformation, the initial void regions regain intimate contact between Li/SE, which then can also experience LBL-like stripping under stack pressure. In this case, a disordered layer is expected to form in the new interface, and the Li metal above, regardless of its crystallinity, would be stripped homogeneously through this disordered layer. In fact, there are two major ways for the Li anode to thin down: (1) void growth and collapse, *via* TPB dissolution and Li creep, respectively; (2) LBL stripping, *via* homogeneous interface dissolution. If the voids are sufficiently suppressed and free surfaces and grain boundaries in Li metal are also annihilated under high stack pressure, the anode thinning should be mainly achieved by LBL(-like) stripping.

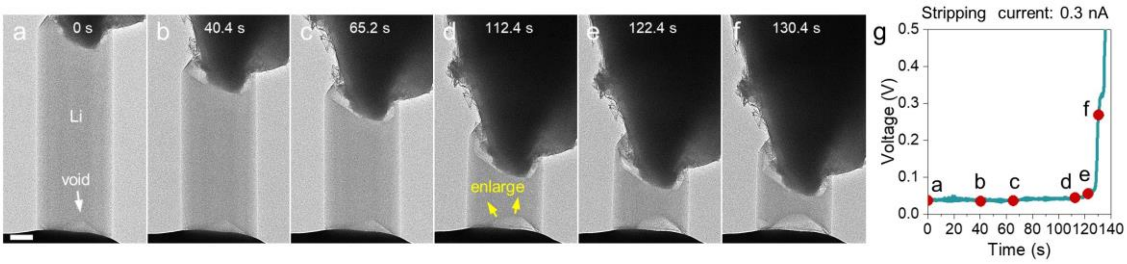

Fig. S28. Void evolution during Li stripping under a decreasing pressure. (a-f) Sequential TEM images showing the Li whisker stripping process. The initial stack pressure was ~7 MPa and the current density was 20 mA cm^{-2} (k=2.8 N m^{-1}). (a-c) In the early stage, the small void remained unchanged in size, resulting in a steady polarization voltage curve in (g). (d-f) As the compressive force from the AFM cantilever decreased with the shortening of the whisker, it cannot inhibit the expansion of void. Therefore, the polarization voltage sharply increased. Scale bar, 500 nm.

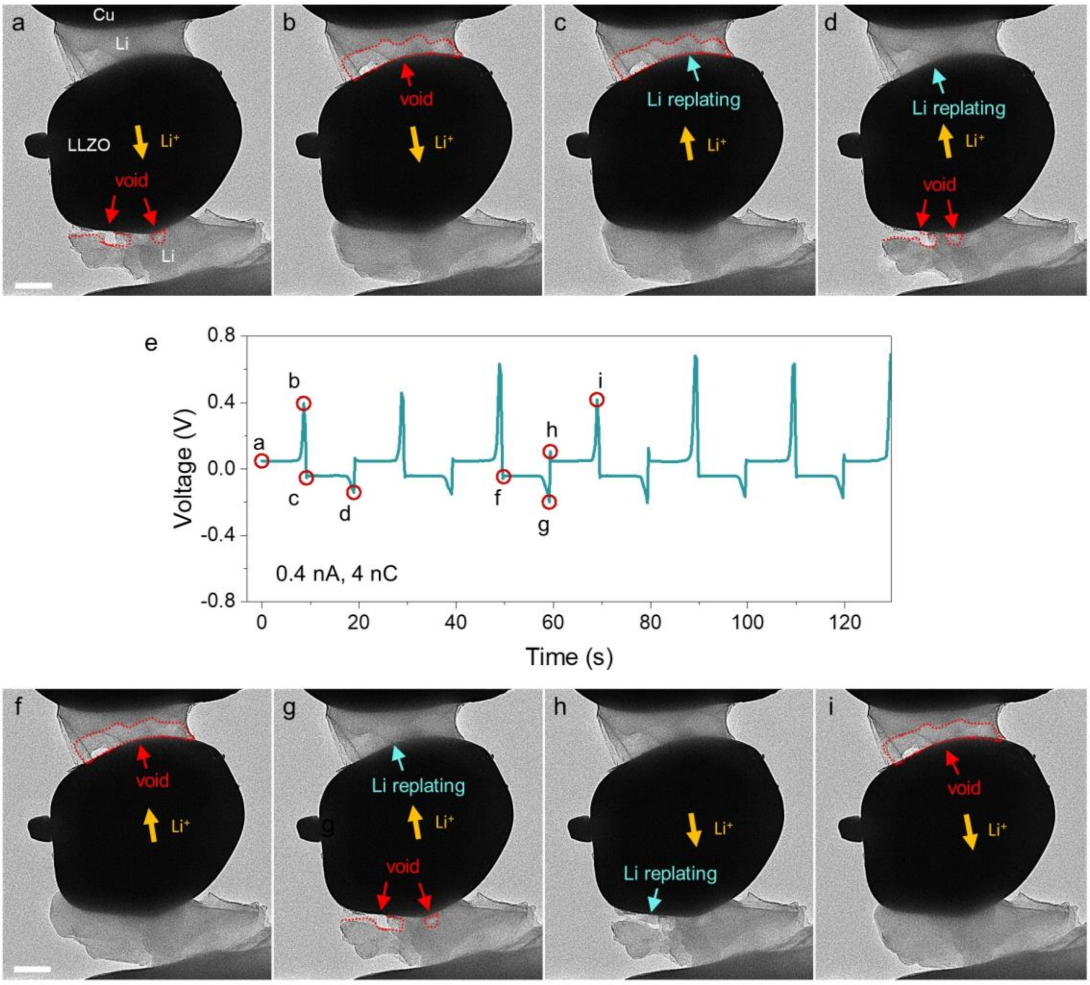

Fig. S29. Galvanostatic cycle of symmetric Li|LLZO|Li microbattery. (a-d) Sequential TEM snapshots from the first cycle of voltage curve in (e). (e) Voltage curve with polarization peaks on the two sides. (f-i) Sequential TEM snapshots of one cycle corresponding to the time points in (e). Scale bars, 500 nm.

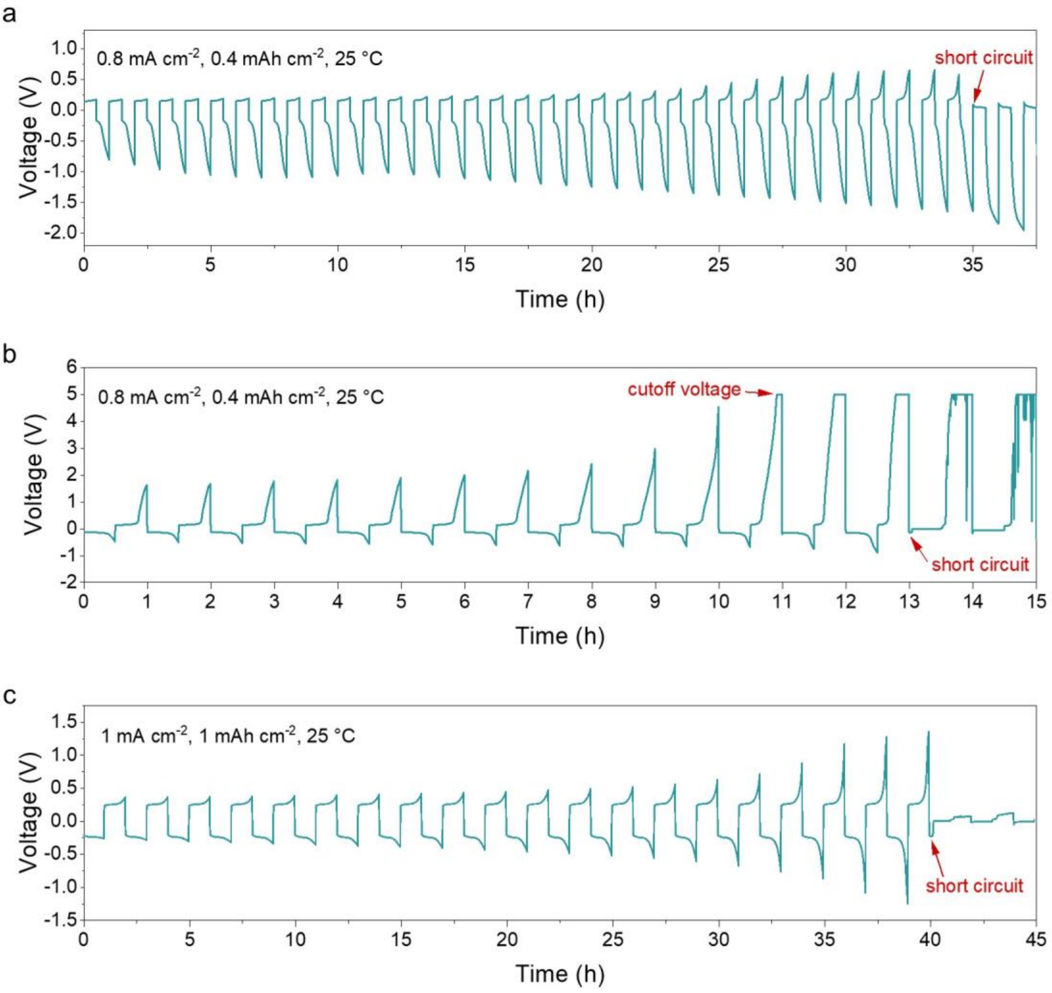

Fig. S30. Voltage profiles of symmetric Li|LLZO|Li bulk cells under galvanostatic cycling with increasing polarization. (a) Cycle curve exhibiting polarization peaks on one side, followed by the gradual emergence of polarization peaks on the other side. Eventually, a short circuit occurred with a sudden drop in voltage. (b) Galvanostatic cycle curve displaying noticeable polarization peaks on both sides, indicative of void formation at the two Li/LLZO interfaces. (c) Voltage profile showing intensified polarization peaks with repeated cycling, suggesting the deterioration of both interfaces due to contact loss. Subsequently, a short circuit happened, indicating the Li dendrites penetration through the LLZO.

3. Movies S1 to S14

Movie S1

In situ TEM observation of Li stripping at 0.2 nA with the mechanical resistance from a rigid Cu CC (Fig. 1A). (Displayed with 1× speed)

Movie S2

In situ TEM observation of Li dissolution at 1 nA with a soft CNT as CC (Fig. 1B). (Displayed with 1× speed)

Movie S3

MD simulation result of homogeneous Li stripping free of constraint (Fig. 1E and fig. S9). (Displayed with 1× speed)

Movie S4

MD simulation result (from a cross section) of void formation under a fixed constraint which impedes the drift of Li metal (fig. S10). (Displayed with 1× speed)

Movie S5

In situ TEM observation of Li stripping under stack pressure exerted by an AFM cantilever (Fig. 3B). (Displayed with 4× speed)

Movie S6

In situ TEM observation of the void annihilated *via* layer-by-layer stripping (Fig. 3C). (Displayed with 1× speed)

Movie S7

In situ TEM observation of void growth suppression during stripping under stack pressure (Fig. 3E). (Displayed with 4× speed)

Movie S8

Phase field simulation results of void evolutions at different current densities under a stack pressure of 10 MPa, including void growth, stabilization and shrinkage, respectively (Fig. 3I). (Displayed

with 1× speed)

Movie S9

In situ TEM observation of interfacial evolution of a Li|LLZO|Li symmetric microbattery during a single galvanostatic cycle (Fig. 4). (Displayed with 1× speed)

Movie S10

In situ TEM observation of the void growth and refilling at a same side of interface of a Li|LLZO|Li symmetric microbattery during six galvanostatic cycles (Fig. 5, A to F). (Displayed with 1× speed)

Movie S11

In situ TEM observation of the void-free cycles of a Li|LLZO|Li symmetric microbattery with the LLZO particle floating up and down (Fig. 5, H to K). (Displayed with 1× speed)

Movie S12

In situ TEM observation of the void-free cycles of a microbattery with CNT as CC (Fig. 5, M to P). (Displayed with 1× speed)

Movie S13

In situ TEM observation of the retraction of Li metal with a high stripping rate of ~2 A cm^{-2} (fig. S7). (Displayed with 1× speed)

Movie S14

In situ TEM observation of the void growth and refilling at two sides of interfaces of a Li|LLZO|Li symmetric microbattery during six galvanostatic cycles (fig. S29). (Displayed with 4× speed)

REFERENCES AND NOTES

1. J. Janek, W. G. Zeier, Challenges in speeding up solid-state battery development. *Nat. Energy* **8**, 230–240 (2023).

2. T. Krauskopf, F. H. Richter, W. G. Zeier, J. Janek, Physicochemical concepts of the lithium metal anode in solid-state batteries. *Chem. Rev.* **120**, 7745–7794 (2020).

3. Y. Cheng, L. Zhang, Q. Zhang, J. Li, Y. Tang, C. Delmas, T. Zhu, M. Winter, M.-S. Wang, J. Huang, Understanding all solid-state lithium batteries through in situ transmission electron microscopy. *Mater. Today* **42**, 137–161 (2021).

4. Z. Ning, D. S. Jolly, G. Li, R. De Meyere, S. D. Pu, Y. Chen, J. Kasemchainan, J. Ihli, C. Gong, B. Liu, D. L. R. Melvin, A. Bonnin, O. Magdysyuk, P. Adamson, G. O. Hartley, C. W. Monroe, T. J. Marrow, P. G. Bruce, Visualizing plating-induced cracking in lithium-anode solid-electrolyte cells. *Nat. Mater.* **20**, 1121–1129 (2021).

5. J. Kasemchainan, S. Zekoll, D. Spencer Jolly, Z. Ning, G. O. Hartley, J. Marrow, P. G. Bruce, Critical stripping current leads to dendrite formation on plating in lithium anode solid electrolyte cells. *Nat. Mater.* **18**, 1105–1111 (2019).

6. E. Kazyak, R. Garcia-Mendez, W. S. LePage, A. Sharafi, A. L. Davis, A. J. Sanchez, K.-H. Chen, C. Haslam, J. Sakamoto, N. P. Dasgupta, Li penetration in ceramic solid electrolytes: *Operando* microscopy analysis of morphology, propagation, and reversibility. *Matter* **2**, 1025–1048 (2020).

7. M. J. Wang, R. Choudhury, J. Sakamoto, Characterizing the Li-solid-electrolyte interface dynamics as a function of stack pressure and current density. *Joule* **3**, 2165–2178 (2019).

8. T. Krauskopf, H. Hartmann, W. G. Zeier, J. Janek, Toward a fundamental understanding of the lithium metal anode in solid-state batteries-An electrochemo-mechanical study on the garnet-type solid electrolyte $Li_{6.25}Al_{0.25}La_3Zr_2O_{12}$. *ACS Appl. Mater. Interfaces* **11**, 14463–14477 (2019).

9. H. Gao, X. Ai, H. Wang, W. Li, P. Wei, Y. Cheng, S. Gui, H. Yang, Y. Yang, M.-S. Wang, Visualizing the failure of solid electrolyte under GPa-level interface stress induced by lithium eruption. *Nat. Commun.* **13**, 5050 (2022).

10. M. Yang, Y. Liu, A. M. Nolan, Y. Mo, Interfacial atomistic mechanisms of lithium metal stripping and plating in solid-state batteries. *Adv. Mater.* **33**, e2008081 (2021).

11. Y. Cheng, Z. Cai, J. Xu, Z. Sun, X. Wu, J. Han, Y.-H. Wang, M.-S. Wang, Zwitterionic cellulose-based polymer electrolyte enabled by aqueous solution casting for high-performance solid-state batteries. *Angew. Chem. Int. Ed.* **63**, e202400477 (2024).

12. Z. Ning, G. Li, D. L. R. Melvin, Y. Chen, J. Bu, D. Spencer-Jolly, J. Liu, B. Hu, X. Gao, J. Perera, C. Gong, S. D. Pu, S. Zhang, B. Liu, G. O. Hartley, A. J. Bodey, R. I. Todd, P. S. Grant, D. E. J. Armstrong, T. J. Marrow, C. W. Monroe, P. G. Bruce, Dendrite initiation and propagation in lithium metal solid-state batteries. *Nature* **618**, 287–293 (2023).

13. C. Lee, S. Y. Han, J. A. Lewis, P. P. Shetty, D. Yeh, Y. Liu, E. Klein, H. W. Lee, M. T. McDowell, Stack pressure measurements to probe the evolution of the lithium-solid-state electrolyte interface. *ACS Energy Lett.* **6**, 3261–3269 (2021).

14. Y. Liu, J. Zhang, B. Zhang, H. Gao, D. Chen, M. Wang, C. Wang, Stability criterion for electrodeposition in solid-state batteries with metallic anodes. *PRX Energy* **3**, 013010 (2024).

15. T. Famprikis, P. Canepa, J. A. Dawson, M. S. Islam, C. Masquelier, Fundamentals of inorganic solid-state electrolytes for batteries. *Nat. Mater.* **18**, 1278–1291 (2019).

16. J. A. Lewis, F. J. Q. Cortes, Y. Liu, J. C. Miers, A. Verma, B. S. Vishnugopi, J. Tippens, D. Prakash, T. S. Marchese, S. Y. Han, C. Lee, P. P. Shetty, H. W. Lee, P. Shevchenko, F. De Carlo, C. Saldana, P. P. Mukherjee, M. T. McDowell, Linking void and interphase evolution to electrochemistry in solid-state batteries using operando X-ray tomography. *Nat. Mater.* **20**, 503–510 (2021).

17. Y. Lu, C.-Z. Zhao, J.-K. Hu, S. Sun, H. Yuan, Z.-H. Fu, X. Chen, J.-Q. Huang, M. Ouyang, Q. Zhang, The void formation behaviors in working solid-state Li metal batteries. *Sci. Adv.* **8**, eadd0510 (2022).

18. K. Lee, E. Kazyak, M. J. Wang, N. P. Dasgupta, J. Sakamoto, Analyzing void formation and rewetting of thin *in situ*-formed Li anodes on LLZO. *Joule* **6**, 2547–2565 (2022).

19. D. Cheng, T. Wynn, B. Lu, M. Marple, B. Han, R. Shimizu, B. Sreenarayanan, J. Bickel, P. Hosemann, Y. Yang, H. Nguyen, W. Li, G. Zhu, M. Zhang, Y. S. Meng, A free-standing lithium phosphorus oxynitride thin film electrolyte promotes uniformly dense lithium metal deposition with no external pressure. *Nat. Nanotechnol.* **18**, 1448–1455 (2023).

20. W. Chang, R. May, M. Wang, G. Thorsteinsson, J. Sakamoto, L. Marbella, D. Steingart, Evolving contact mechanics and microstructure formation dynamics of the lithium metal-$Li_7La_3Zr_2O_{12}$ interface. *Nat. Commun.* **12**, 6369 (2021).

21. F. Yonemoto, A. Nishimura, M. Motoyama, N. Tsuchimine, S. Kobayashi, Y. Iriyama, Temperature effects on cycling stability of Li plating/stripping on Ta-doped $Li_7La_3Zr_2O_{12}$. *J. Power Sources* **343**, 207–215 (2017).

22. V. Raj, V. Venturi, V. R. Kankanallu, B. Kuiri, V. Viswanathan, N. P. B. Aetukuri, Direct correlation between void formation and lithium dendrite growth in solid-state electrolytes with interlayers. *Nat. Mater.* **21**, 1050–1056 (2022).

23. D. S. Jolly, Z. Ning, J. E. Darnbrough, J. Kasemchainan, G. O. Hartley, P. Adamson, D. E. J. Armstrong, J. Marrow, P. G. Bruce, Sodium/Na β alumina interface: Effect of pressure on voids. *ACS Appl. Mater. Interfaces* **12**, 678–685 (2020).

24. F. Sagane, R. Shimokawa, H. Sano, H. Sakaebe, Y. Iriyama, In-situ scanning electron microscopy observations of Li plating and stripping reactions at the lithium phosphorus oxynitride glass electrolyte/Cu interface. *J. Power Sources* **225**, 245–250 (2013).

25. T. Krauskopf, B. Mogwitz, H. Hartmann, D. K. Singh, W. G. Zeier, J. Janek, The fast charge transfer kinetics of the lithium metal anode on the garnet-type solid electrolyte $Li_{6.25}Al_{0.25}La_3Zr_2O_{12}$. *Adv. Energy Mater.* **10**, 2000945 (2020).

26. M. Jäckle, K. Helmbrecht, M. Smits, D. Stottmeister, A. Groß, Self-diffusion barriers: Possible descriptors for dendrite growth in batteries? *Energ. Environ. Sci.* **11**, 3400–3407 (2018).

27. T. Krauskopf, R. Dippel, H. Hartmann, K. Peppler, B. Mogwitz, F. H. Richter, W. G. Zeier, J. Janek, Lithium-metal growth kinetics on LLZO garnet-type solid electrolytes-*Operando* study of lithium deposition and dendrite growth. *Joule* **3**, 2030–2049 (2019).

28. Y. Chen, Z. Wang, X. Li, X. Yao, C. Wang, Y. Li, W. Xue, D. Yu, S. Y. Kim, F. Yang, A. Kushima, G. Zhang, H. Huang, N. Wu, Y. W. Mai, J. B. Goodenough, J. Li, Li metal deposition and stripping in a solid-state battery via Coble creep. *Nature* **578**, 251–255 (2020).

29. S. Yu, D. J. Siegel, Grain Boundary contributions to Li-ion transport in the solid electrolyte $Li_7La_3Zr_2O_{12}$ (LLZO). *Chem. Mater.* **29**, 9639–9647 (2017).

30. B. Gao, R. Jalem, Y. Tateyama, Atomistic insight into the dopant impacts at the garnet $Li_7La_3Zr_2O_{12}$ solid electrolyte grain boundaries. *J. Mater. Chem. A* **10**, 10083–10091 (2022).

31. B. Gao, R. Jalem, H.-K. Tian, Y. Tateyama, Revealing atomic-scale ionic stability and transport around grain boundaries of garnet $Li_7La_3Zr_2O_{12}$ solid electrolyte. *Adv. Energy Mater.* **12**, 2102151 (2022).

32. R. Raj, Nucleation of voids at Li-metal–ceramic–electrolyte interfaces. *MRS Commun.* **11**, 644–649 (2021).

33. K. Lee, J. Sakamoto, Li stripping behavior of anode-free solid-state batteries under intermittent-current discharge conditions. *Adv. Energy Mater.* **14**, 2303571 (2024).

34. T. Swamy, R. Park, B. W. Sheldon, D. Rettenwander, L. Porz, S. Berendts, R. Uecker, W. C. Carter, Y. M. Chiang, Lithium metal penetration induced by electrodeposition through solid

electrolytes: Example in single-crystal $Li_6La_3ZrTaO_{12}$ garnet. *J. Electrochem. Soc.* **165**, A3648-A3655 (2018).

35. R. Choudhury, M. Wang, J. Sakamoto, The effects of electric field distribution on the interface stability in solid electrolytes. *J. Electrochem. Soc.* **167**, 140501 (2020).

36. H.-K. Tian, B. Xu, Y. Qi, Computational study of lithium nucleation tendency in $Li_7La_3Zr_2O_{12}$ (LLZO) and rational design of interlayer materials to prevent lithium dendrites. *J. Power Sources* **392**, 79–86 (2018).

37. H. C. Wang, H. W. Gao, X. X. Chen, J. P. Zhu, W. Q. Li, Z. L. Gong, Y. X. Li, M. S. Wang, Y. Yang, Linking the defects to the formation and growth of Li dendrite in all-solid-state batteries. *Adv. Energy Mater.* **11**, 2102148 (2021).

38. C. Xu, Z. Ahmad, A. Aryanfar, V. Viswanathan, J. R. Greer, Enhanced strength and temperature dependence of mechanical properties of Li at small scales and its implications for Li metal anodes. *Proc. Natl. Acad. Sci. U.S.A.* **114**, 57–61 (2017).

39. Y. He, X. D. Ren, Y. B. Xu, M. H. Engelhard, X. L. Li, J. Xiao, J. Liu, J. G. Zhang, W. Xu, C. M. Wang, Origin of lithium whisker formation and growth under stress. *Nat. Nanotechnol.* **14**, 1042–1047 (2019).

40. L. Zhang, T. Yang, C. Du, Q. Liu, Y. Tang, J. Zhao, B. Wang, T. Chen, Y. Sun, P. Jia, H. Li, L. Geng, J. Chen, H. Ye, Z. Wang, Y. Li, H. Sun, X. Li, Q. Dai, Y. Tang, Q. Peng, T. Shen, S. Zhang, T. Zhu, J. Huang, Lithium whisker growth and stress generation in an in situ atomic force microscope-environmental transmission electron microscope set-up. *Nat. Nanotechnol.* **15**, 94–98 (2020).

41. H. Yan, K. Tantratian, K. Ellwood, E. T. Harrison, M. Nichols, X. Cui, L. Chen, How does the creep stress regulate void formation at the lithium-solid electrolyte interface during stripping? *Adv. Energy Mater.* **12**, 2102283 (2022).

42. E. Kazyak, M. J. Wang, K. Lee, S. Yadavalli, A. J. Sanchez, M. D. Thouless, J. Sakamoto, N. P. Dasgupta, Understanding the electro-chemo-mechanics of Li plating in anode-free solid-state batteries with *operando* 3D microscopy. *Matter* **5**, 3912–3934 (2022).

43. D. K. Singh, T. Fuchs, C. Krempaszky, P. Schweitzer, C. Lerch, F. H. Richter, J. Janek, Origin of the lithium metal anode instability in solid-state batteries during discharge. *Matter* **6**, 1463–1483 (2023).

44. E. G. Herbert, S. A. Hackney, V. Thole, N. J. Dudney, P. S. Phani, Nanoindentation of high-purity vapor deposited lithium films: A mechanistic rationalization of diffusion-mediated flow. *J. Mater. Res.* **33**, 1347–1360 (2018).

45. A. Masias, N. Felten, R. Garcia-Mendez, J. Wolfenstine, J. Sakamoto, Elastic, plastic, and creep mechanical properties of lithium metal. *J. Mater. Sci.* **54**, 2585–2600 (2019).

46. W. S. LePage, Y. X. Chen, E. Kazyak, K. H. Chen, A. J. Sanchez, A. Poli, E. M. Arruda, M. D. Thouless, N. P. Dasgupta, Lithium mechanics: Roles of strain rate and temperature and implications for lithium metal batteries. *J. Electrochem. Soc.* **166**, A89–A97 (2019).

47. J. A. B. Agier, S. S. Shishvan, N. A. Fleck, V. S. Deshpande, Void growth within Li electrodes in solid electrolyte cells. *Acta Mater.* **240**, 118303 (2022).

48. X. Zhang, Q. J. Wang, K. L. Harrison, S. A. Roberts, S. J. Harris, Pressure-driven interface evolution in solid-state lithium metal batteries. *Cell Rep. Phys. Sci.* **1**, 100012 (2020).

49. D. K. Singh, T. Fuchs, C. Krempaszky, B. Mogwitz, S. Burkhardt, F. H. Richter, J. Janek, Overcoming anode instability in solid-state batteries through control of the lithium metal microstructure. *Adv. Funct. Mater.* **33**, 2211067 (2023).

50. M. J. Wang, E. Kazyak, N. P. Dasgupta, J. Sakamoto, Transitioning solid-state batteries from lab to market: Linking electro-chemo-mechanics with practical considerations. *Joule* **5**, 1371–1390 (2021).

51. T. Fuchs, C. G. Haslam, A. C. Moy, C. Lerch, T. Krauskopf, J. Sakamoto, F. H. Richter, J. Janek, Increasing the pressure-free stripping capacity of the lithium metal anode in solid-state-batteries by carbon nanotubes. *Adv. Energy Mater.* **12**, 2201125 (2022).

52. M. Zhu, O. G. Schmidt, Tiny robots and sensors need tiny batteries—Here’s how to do it. *Nature* **589**, 195–197 (2021).

53. A. Verma, J. B. Singh, S. D. Kaushik, V. Siruguri, Lattice parameter variation and its effect on precipitation behaviour of ordered Ni_2(Cr,Mo) phase in Ni-Cr-Mo alloys. *J. Alloys Compd.* **813**, 152195 (2020).

54. H. Haftbaradaran, J. Song, W. A. Curtin, H. Gao, Continuum and atomistic models of strongly coupled diffusion, stress, and solute concentration. *J. Power Sources* **196**, 361–370 (2011).

55. D. Hull, D. E. Rimmer, The growth of grain-boundary voids under stress. *Philos. Mag.* **4**, 673–687 (1959).

56. Y. Zhao, R. Wang, E. Martínez-Pañeda, A phase field electro-chemo-mechanical formulation for predicting void evolution at the Li–electrolyte interface in all-solid-state batteries. *J. Mech. Phys. Solids* **167**, 104999 (2022).

57. J. Kundin, R. Siquieri, H. Emmerich, A quantitative multi-phase-field modeling of the microstructure evolution in a peritectic Al–Ni alloy. *Physica D* **243**, 116–127 (2013).

58. K. Ammar, B. Appolaire, G. Cailletaud, F. Feyel, S. Forest, Finite element formulation of a phase field model based on the concept of generalized stresses. *Comput. Mater. Sci.* **45**, 800–805 (2009).

59. R. Folch, M. Plapp, Quantitative phase-field modeling of two-phase growth. *Phys. Rev. E* **72**, 011602 (2005).

60. S.-L. Wang, R. F. Sekerka, A. A. Wheeler, B. T. Murray, S. R. Coriell, R. J. Braun, G. B. McFadden, Thermodynamically-consistent phase-field models for solidification. *Physica D* **69**, 189–200 (1993).

61. M. Z. Bazant, Theory of chemical kinetics and charge transfer based on nonequilibrium thermodynamics. *Acc. Chem. Res.* **46**, 1144–1160 (2013).

62. S. G. Kim, W. T. Kim, T. Suzuki, Phase-field model for binary alloys. *Phys. Rev. E* **60**, 7186–7197 (1999).

63. C. Lin, H. Ruan, Panoramic modeling of lithium dendrite formation and crack penetration in solid-state electrolyte: Mechanism and suppression strategies. *J. Power Sources* **598**, 234137 (2024).

64. R. Zhang, X. Shen, Y.-T. Zhang, X.-L. Zhong, H.-T. Ju, T.-X. Huang, X. Chen, J.-D. Zhang, J.-Q. Huang, Dead lithium formation in lithium metal batteries: A phase field model. *J. Energy Chem.* **71**, 29–35 (2022).

65. H. Schmalzried, J. Janek, Chemical kinetics of phase boundaries in solids. *Ber. Bunsen. Phys. Chem* **102**, 127–143 (1998).

66. M. Wang, J. B. Wolfenstine, J. Sakamoto, Temperature dependent flux balance of the $Li/Li_7La_3Zr_2O_{12}$ interface. *Electrochim. Acta* **296**, 842–847 (2019).

67. A. P. Thompson, H. M. Aktulga, R. Berger, D. S. Bolintineanu, W. M. Brown, P. S. Crozier, P. J. in't Veld, A. Kohlmeyer, S. G. Moore, T. D. Nguyen, R. Shan, M. J. Stevens, J. Tranchida, C. Trott, S. J. Plimpton, LAMMPS—A flexible simulation tool for particle-based materials modeling at the atomic, meso, and continuum scales. *Comput. Phys. Commun.* **271**, 108171 (2022).

68. A. Nichol, G. J. Ackland, Property trends in simple metals: An empirical potential approach. *Phys. Rev. B* **93**, 184101 (2016).

69. M. Yang, Y. Mo, Interfacial defect of lithium metal in solid-state batteries. *Angew. Chem. Int. Ed.* **60**, 21494–21501 (2021).

70. M. Yang, Y. Liu, Y. Mo, Lithium crystallization at solid interfaces. *Nat. Commun.* **14**, 2986 (2023).

71. A. Stukowski, Visualization and analysis of atomistic simulation data with OVITO—The Open Visualization Tool. *Modell. Simul. Mater. Sci. Eng.* **18**, 015012 (2010).
